# Room‐Temperature Alane Dehydrogenation for Visible‐Light‐Driven Photocatalytic Hydrogen Supply System

**DOI:** 10.1002/advs.202518927

**Published:** 2025-12-12

**Authors:** Ting‐Ting Li, Li‐Cheng Liang, Rui‐Qi Chen, Chun‐Qi Zhang, Sheng‐Nan Zhang, Wen‐Wen Cheng, Xi‐Hao Chen, Ning Wang, Juan‐Ding Xiao, Qing‐Qing Yang, Fei Liang, Chao‐Feng Zhang

**Affiliations:** ^1^ Institutes of Physical Science and Information Technology Anhui Graphene Carbon Fiber Materials Research Center Anhui University Hefei 230601 China; ^2^ School of Materials Science and Engineering Anhui Provincial Key Laboratory of Magnetic Functional Materials and Devices Center for Free Electron Laser & High Magnetic Field Anhui University Hefei 230601 China; ^3^ School of Materials Science and Engineering Chongqing University of Arts and Sciences Chongqing 402160 China; ^4^ School of Science Key Laboratory of High Performance Scientific Computation Xihua University Chengdu 610039 China

**Keywords:** Al/MOF heterostructure, alanes, LSPR, non‐thermodynamic mechanism, visible‐light‐driven photocatalysis

## Abstract

Solar‐driven hydrogen supply systems filled with high‐density hydrides can overcome the traditional limitations of external heating and power sources. However, these systems commonly rely on photothermal effects to elevate the hydride surface temperature, significantly restricting their photon‐to‐chemical conversion efficiency. Therefore, exploring hydrogen supply systems driven by visible‐light photocatalysis offers immense potential for achieving enhanced photon‐to‐chemical conversion. In this study, a non‐thermodynamic regulation mechanism based on the dehydrogenation of alane and driven by the broadband‐responsive photocatalysis of AlH_3_‐MOF is investigated. The dehydrogenation rate under visible‐light irradiation reaches 30.8 µmol g^−1^ min^−1^, achieving a better than 20‐fold improvement compared to room‐temperature dark conditions. Moreover, a hydrogen release capacity of 4.7 wt.% is achieved at an ultra‐low light intensity of 0.37 W cm^−2^ without external heating. Experimental investigations confirm the in situ formation of a novel Al/MOF heterostructure during photocatalytic dehydrogenation. Al nanoparticles induce the injection of hot electrons into the MOF via localized surface plasmon resonance, significantly prolonging the photogenerated charge carrier lifetime. Density functional theory calculations reveal that AlH_3_ chemisorption at Al/MOF interfaces induces interfacial charge redistribution and establishes a direct interfacial charge transfer channel. This study pioneers a non‐thermodynamic photocatalytic regulation paradigm for solid‐state high‐energy hydrides, enabling portable application in abundant solar‐irradiated regions.

## Graphical abstract

In situ formed Al/MOF enables viable visible‐light‐driven photocatalytic dehydrogenation of alane (AlH_3_) confirming practical application. Results demonstrate that Al nanoparticles prolong lifetime of photogenerated charge carriers via localized surface plasmon resonance induced hot electron injection, while AlH_3_ chemisorption triggers interfacial charge redistribution to accelerate Al─H bond cleavage.

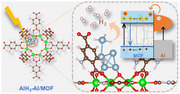

## 1 Introduction

The high energy density of hydrogen demonstrates its suitability as an ideal renewable energy carrier.^[^ ^1^, ^2^, ^3^ ^]^ Solid‐state hydrogen storage systems demonstrate substantial engineering potential due to their high storage density, high cycling reversibility, and inherent safety.^[^ ^4^, ^5^, ^6^ ^]^ Among the various high‐density hydrides, alane (AlH_3_) demonstrates exceptional gravimetric (10.1 wt.%) and volumetric (148 kg m^−3^) hydrogen capacities.^[^ ^7^ ^]^ However, the thermodynamic stability of AlH_3_ means that this material exhibits a high dehydrogenation temperature, which typically ranges from 150 to 200 °C.^[^ ^8^ ^]^ The dehydrogenation of hydrides generally requires external electric heating, which lowers the overall energy density of hydrogen storage systems and increases their costs for further applications.

Benefiting from its abundance and renewability, solar energy has emerged as a viable alternative to fossil fuels in sustainable energy systems.^[^ ^9^ ^]^ Utilizing solar‐driven systems to supply hydrogen energy offers a new paradigm for breaking the limitations of traditional electric heating and power sources, enabling the controllable release of hydrogen from solid‐state storage systems without external heating devices. The photothermal‐catalytic synergistic hydrogen supply strategy developed by Yu et al.^[^ ^10^, ^11^, ^12^, ^13^ ^]^ innovatively integrates localized photothermal effects with interfacial catalysis. This system elevates the hydride surface temperature (exceeding 120 °C) without external heating, while catalytic sites weaken the metal hydrogen bonds to achieve the dehydrogenation of the hydrides. Notably, while this strategy circumvents energy consumption from electric heating, the multi‐stage energy conversion pathway (photon energy → thermal energy → chemical energy) still suffers from multi‐step energy losses. Compared to photothermal effects, photocatalysis enables the direct generation of photoexcited high‐energy charge carriers, which subsequently participate in redox reactions.^[^ ^14^ ^]^ This approach enables more efficient energy conversion and allows reactions to spontaneously proceed under mild conditions. Chen et al.^[^ ^15^, ^16^ ^]^ and Xia et al.^[^ ^17^ ^]^ have demonstrated that lithium hydride under Ultraviolet‐Visible (UV–vis) illumination can generate long‐lived photoexcited electrons localized with hydrogen vacancies. Gabis et al.^[^ ^18^, ^19^, ^20^ ^]^ elucidated a UV‐activated dehydrogenation mechanism of AlH_3_ through a combination of experimental investigations and density functional theory (DFT) calculations. They demonstrated that UV irradiation induces the formation of hydrogen vacancies within AlH_3_, providing nucleation sites for metallic phase formation. However, the UV spectral region only accounts for 4% of the total solar spectrum,^[^ ^21^ ^]^ which fundamentally constrains the photon‐to‐chemical energy conversion efficiency of this UV‐activated process. Therefore, constructing a broadband‐responsive photocatalytic system would lead to more efficient visible light harvesting, enhancing the photon‐to‐chemical energy conversion efficiency. This would enable the highly efficient dehydrogenation of hydrides at room temperature and even lower ambient temperatures.

Metal‐organic framework (MOF)‐based semiconductor photocatalysts offer advantages such as large surface areas and abundant catalytically active sites.^[^ ^22^, ^23^, ^24^ ^]^ Several strategies have been adopted to improve the photocatalytic properties of MOFs. For example, MOF composites incorporating plasmonic metals such as Pd, Pt, or Au exhibit broadly tunable localized surface plasmon resonance (LSPR) properties.^[^ ^25^, ^26^, ^27^ ^]^ In plasmonic metal/MOF photocatalysts, LSPR improves the photocatalytic activity of the MOF through light scattering, hot electron, near‐field enhancement, and photothermal effects.^[^ ^28^, ^29^, ^30^, ^31^, ^32^ ^]^ However, while noble metal plasmonic materials (e.g., Au, Ag) demonstrate excellent performance, their low natural abundance and high cost significantly hinder practical applications. In contrast, aluminum (Al), which is the most abundant metallic element in the earth's crust can function as an efficient plasmonic support material,^[^ ^33^ ^]^ exhibiting broadband light absorption and superior work function alignment with common semiconductor band structures.^[^ ^34^ ^]^ Therefore, constructing a heterostructure between MOF and Al nanoparticles (NPs) is expected to provide improved light‐harvesting properties and enhanced charge‐separation efficiency, boosting the photocatalytic dehydrogenation performance for high‐energy hydrides.

**Figure 1:**
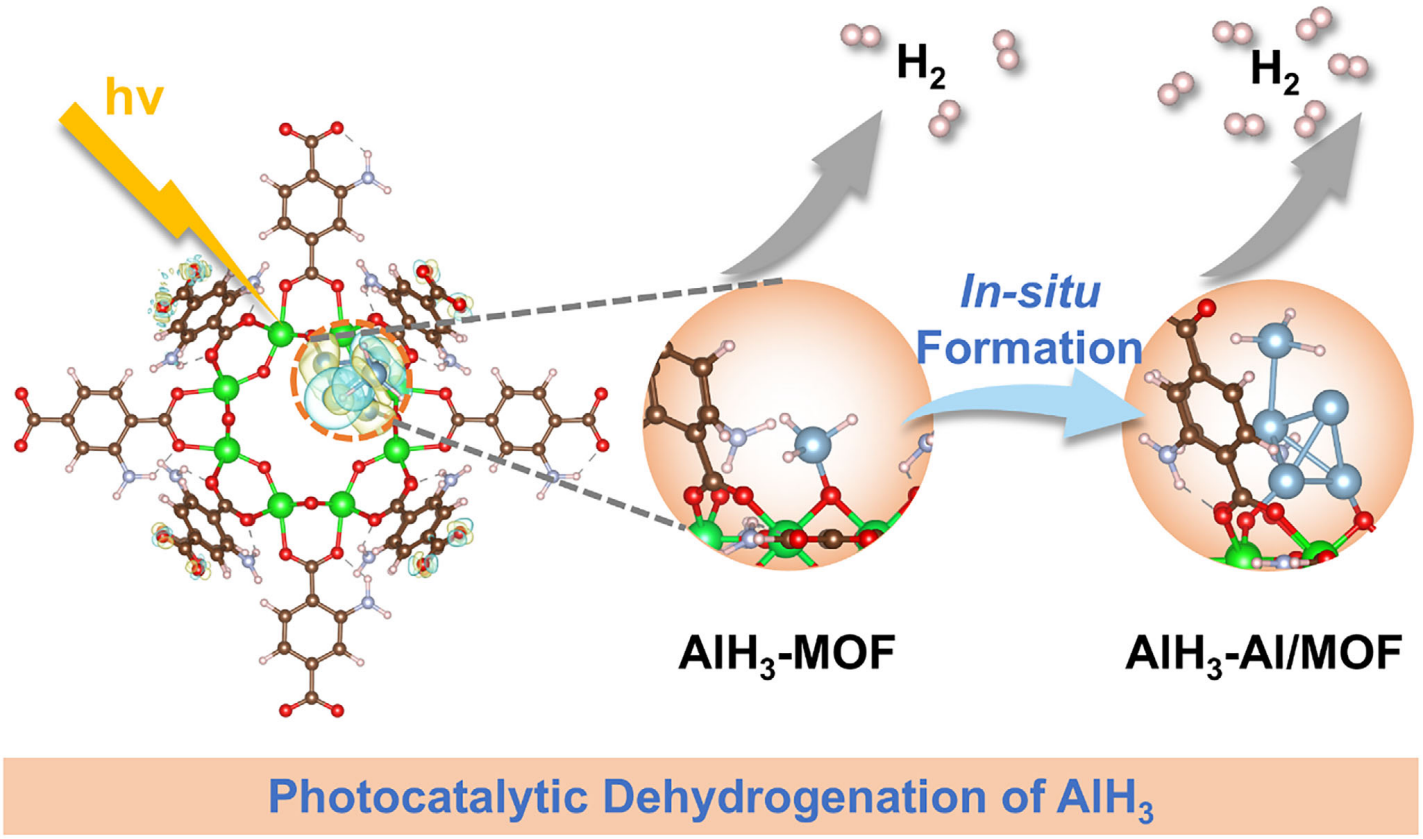
Schematic illustration of this study. Visible‐light‐driven photocatalytic dehydrogenation mechanism of AlH_3_.

The amino (−NH_2_) functional group can extend the optical absorption range of MOFs to the visible light region, thereby improving their visible light‐driven photocatalytic activity.^[^ ^35^ ^]^ Therefore, a broadband‐responsive photocatalytic system composited of AlH_3_ and a MOF (NH_2_‐MIL‐125) is designed and constructed to achieve the efficient visible‐light‐driven photocatalytic dehydrogenation of AlH_3_ at room temperature via the in situ formation of an Al/MOF heterostructure (**Figure** **1**). Plasmonic Al NPs extend the optical absorption range of the MOF in the visible region, and LSPR‐induced hot electron injection significantly prolongs the lifetime of the charge carriers. Complementary DFT calculations reveal that the chemisorption of AlH_3_ at Al/MOF interfaces induces interfacial charge redistribution and establishes a direct interfacial charge transfer channel, expediting the breaking of Al─H bonds. At room temperature under an ultra‐low light intensity of 0.37 W cm^−2^, the visible‐light‐driven dehydrogenation rate reaches 30.8 µmol g^−1^ min^−1^, which is a 20.5‐fold improvement compared to dark conditions. In addition, AlH_3_‐MOF achieves a visible‐light‐driven hydrogen release capacity of 4.7 wt.% within 180 min at a near‐ambient temperature without additional heating devices. This study reveals a non‐thermodynamic photocatalytically driven regulation pathway for solid‐state hydrogen storage materials for the first time. By harnessing photogenerated charges to directly manipulate the reaction at the electronic level, this pathway transcends conventional energy‐input modes and offers a new opportunity for high‐energy hydrides. Furthermore, by enabling the photocatalytic dehydrogenation of hydrides under an ultra‐low light intensity at room temperature, this work represents a substantial step forward for the practical application of solar‐driven hydrogen supply systems in abundantly solar‐irradiated regions and frigid areas.

## 2 Results

### 2.1 Electronic and Optical Properties of α‐AlH_3_

**Figure 2:**
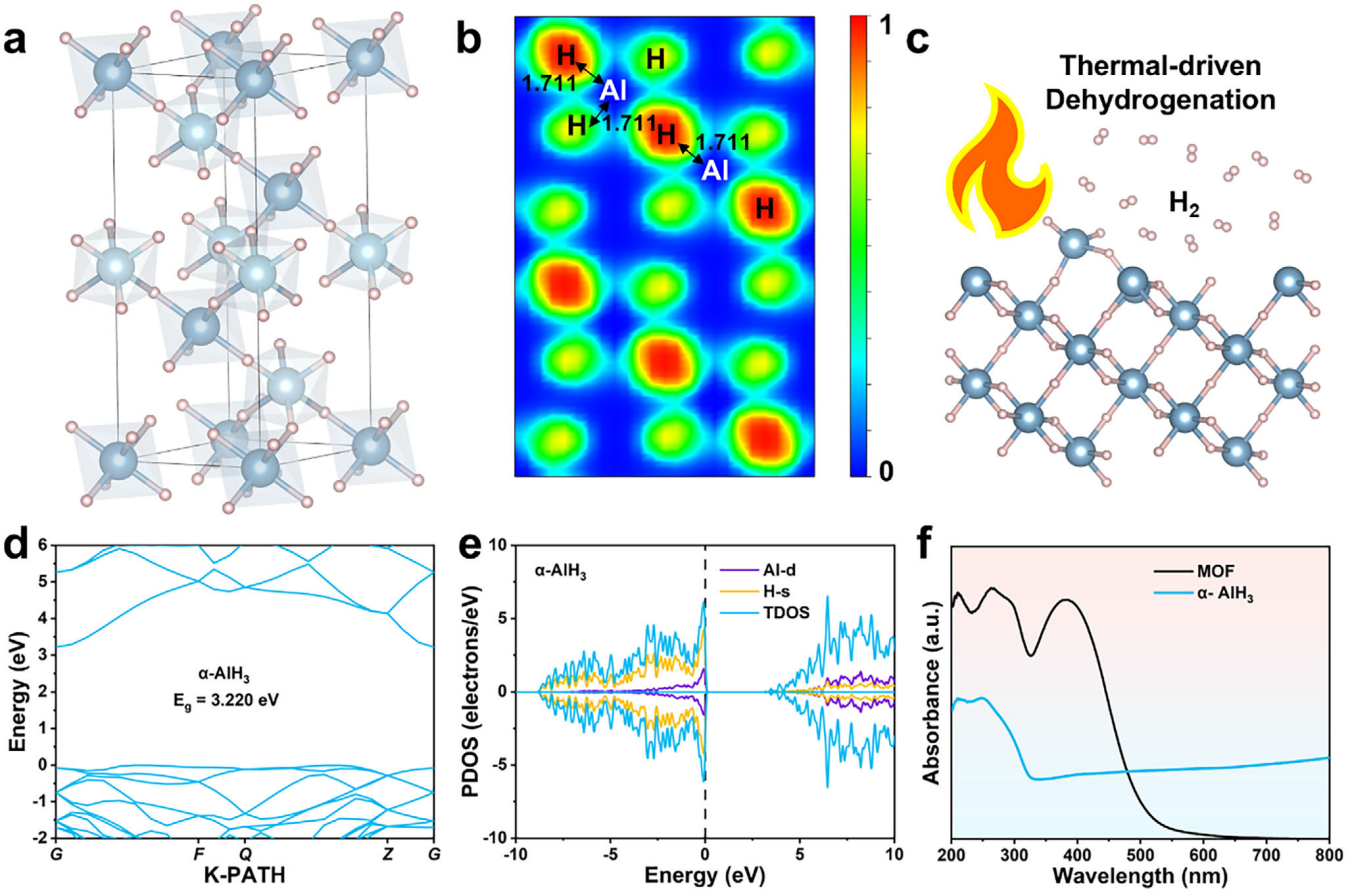
Electronic and optical properties of α‐AlH_3_. a) Crystal structure and b) charge density plots of α‐AlH_3_. c) Schematic illustration of conventional thermally driven dehydrogenation of α‐AlH_3_. d) Band structure and e) calculated DOS of α‐AlH_3_. f) UV–vis absorption spectra of α‐AlH_3_ and MOF. Source data are provided as a source data file.

The crystal structure of α‐AlH_3_, which has a hexagonal unit cell, is displayed in **Figure** **2**a. Within α‐AlH_3_, the interatomic distance of the Al─H bond is precisely calculated as 1.711 Å (Figure 2b), consistent with the reported value of 1.715 Å (Ref.[36]). Current studies have confirmed that AlH_3_ exists in at least seven polymorphs α, α', β, γ, δ, ε, and ζ.^[^ ^37^ ^]^ Among these, α‐AlH_3_ is considered to be the most thermodynamically stable form, meaning that α‐AlH_3_ is the predominant candidate for utilization in solid‐state hydrogen storage systems. The conventional dehydrogenation of hydrides relies on external electrical heating to cleave the Al─H bonds and hydrogen through thermally driven pathways, as shown in Figure 2c.

To explore the structural stability and electronic properties of α‐AlH_3_, the band structure and density of states (DOS) of this material were investigated through DFT calculations. Figure 2d presents the calculated band structure of α‐AlH_3_ along the high‐symmetry direction. A direct bandgap of 3.2 eV is observed, unequivocally classifying α‐AlH_3_ as a wide bandgap semiconductor. Complementary DOS analysis of α‐AlH_3_ (Figure 2e) reveals dominant contributions from the H‐*s* orbitals below the Fermi level and Al‐*p* orbitals above the Fermi level. Crucially, the bandgap of 3.2 eV that emerges near the Fermi level arises from energy separation between the H‐*s* state and Al‐*p* state, confirming the semiconductor nature of this material. Furthermore, strong localized hybridization between the Al‐*p* and H‐*s* orbitals forms the Al─H bonds, enhancing structural stability and enabling high‐density hydrogen storage. These intrinsic electronic properties establish α‐AlH_3_ as a promising material for a solid‐state hydrogen supply system.

Notably, the UV–vis absorption spectrum of α‐AlH_3_ exhibits an absorption edge at 333 nm corresponding to an optical bandgap of 3.72 eV (Figures 2f; S1, Supporting Information). This demonstrates that the intrinsic UV light responsiveness (λ <400 nm) of α‐AlH_3_ severely restricts its solar energy‐driven dehydrogenation efficiency.

### 2.2 Preparation and Characterization of AlH_3_‐MOF

To provide enhanced visible light absorption properties, an amino‐functionalized MOF (NH_2_‐MIL‐125) was employed as a representative visible‐light‐responsive photocatalyst. As illustrated in Figure S2a (Supporting Information), NH_2_‐MIL‐125 was synthesized via a solvothermal method with titanium (IV) isopropoxide (TTIP) as the titanium source and 2‐aminoterephthalic acid (NH_2_‐BDC) as an organic linker. The resulting bright yellow powder exhibits a well‐defined cubic morphology as confirmed by scanning electron microscopy (SEM, Figure S2b, Supporting Information). Nitrogen‐adsorption analysis of this MOF at 77 K reveals a Type I adsorption isotherm (Figure S2c, Supporting Information), which is characteristic of microporous materials. Moreover, the MOF has with a Brunauer–Emmett–Teller (BET) surface area of 1205 m^2^ g^−1^. The powder X‐ray diffraction (XRD) pattern of NH_2_‐MIL‐125 (Figure S2d, Supporting Information) precisely matches the simulated pattern, demonstrating phase purity and structural integrity. As displayed in Figures 2f and S1 (Supporting Information), NH_2_‐MIL‐125 demonstrates extended light absorption properties up to 470 nm corresponding to an optical bandgap of 2.64 eV, enabling the highly efficient utilization of visible light (λ >400 nm). These results indicate that a broadband‐responsive photocatalytic material consisting of AlH_3_ and this MOF could enable visible‐light‐driven dehydrogenation beyond the limitations of the full spectrum.

**Figure 3:**
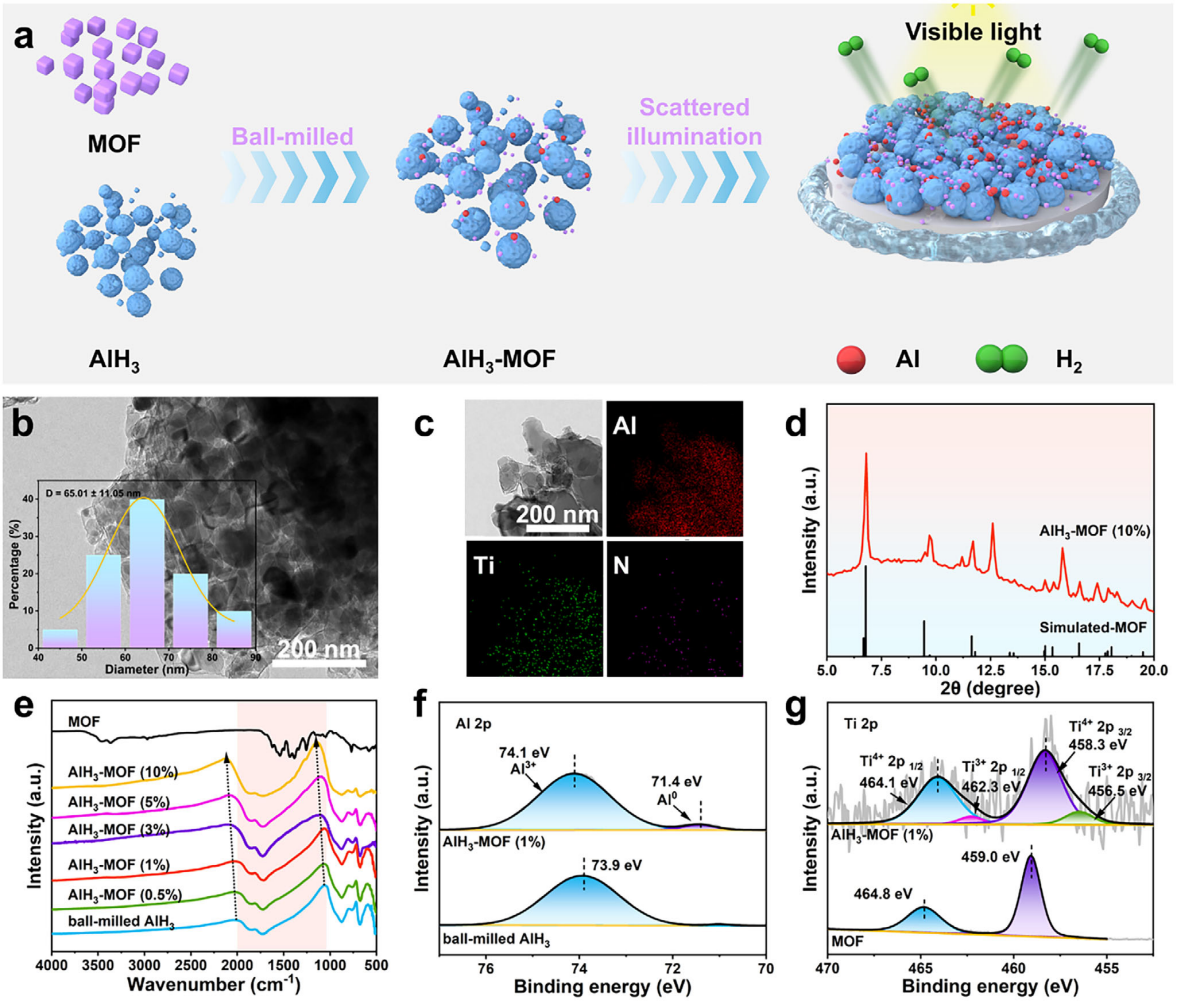
Synthesis and characterization of AlH_3_‐MOF. a) Schematic illustration of the fabrication of AlH_3_‐MOF. b) TEM image and corresponding particle size distribution, and c) TEM‐EDS elemental mapping images of AlH_3_‐MOF (1%). d) Conventional XRD pattern of AlH_3_‐MOF (10%). e) FT‐IR spectra of AlH_3_‐MOF. High‐resolution f) Al 2*p* and g) Ti 2*p* XPS spectra of AlH_3_‐MOF (1%), with the spectra of ball‐milled AlH_3_ and MOF provided for comparison.

A series of AlH_3_‐MOF composites was fabricated by mechanical ball‐milling, as schematically depicted in **Figure** **3**a. Transmission electron microscopy (TEM, Figure 3b) and SEM (Figure S4, Supporting Information) analyses demonstrate that the mechanical ball‐milling process effectively refines the alane grains to 65 nm, exposing the activated crystalline facets of AlH_3_ and enhancing the interfacial contact area with the MOF. Energy‐dispersive X‐ray spectroscopy (EDS) analysis was coupled with TEM to obtain elemental mapping images (Figure 3c), confirming the uniform distribution of Al, Ti, and N throughout the composite as well as the dispersion of the non‐agglomerated MOF around the AlH_3_ phase. This analysis demonstrates that the photocatalyst is uniformly distributed within the composite, providing abundant contact interfaces for photocatalytic dehydrogenation. Additionally, to prevent oxidation and decomposition of AlH_3_, a custom XRD sample holder protected (with Kapton tape) was used to isolate the air. The XRD measurements of composites reveal characteristic diffraction peaks ascribed to AlH_3_ (Figure S5a, Supporting Information), confirming the structural integrity of AlH_3_ during mechanical ball‐milling. However, the Kapton tape on the surface of the custom sample holder produces diffraction peaks at 2θ ≈10–20°, which overlap with and obscure the key diffraction peaks of the MOF in this location (Figure S5b, Supporting Information). To further illustrate the diffraction peaks of MOF, a conventional XRD sample holder was selected for testing. Despite the inevitable atmospheric exposure during this analysis, the characteristic diffraction peaks of the MOF are clearly observed (Figure 3d), confirming the preserved structural integrity of the MOF architecture without framework collapse during mechanical ball‐milling. Therefore, the prepared AlH_3_‐MOF composites maintain MOF‐specific photocatalytic functionalities, including broadband light absorption and abundant active sites, which are critical for enabling the subsequent photocatalytic dehydrogenation process.

Fourier transform infrared (FT‐IR) spectra of AlH_3_‐MOF and the ball‐milled AlH_3_ (Figure 3e) show that the characteristic absorption peaks of AlH_3_ at 677, 760, 874, 1724, and 1843 cm^−1^ are retained after ball‐milling. With increasing MOF loading, a redshift in the Al─H stretching vibrations at 1724 and 1843 cm^−1^ is induced, indicating the weakening of the Al─H bond due to electron density redistribution at the AlH_3_‐MOF interfaces. High‐resolution X‐ray photoelectron spectroscopy (XPS) measurements were performed to further investigate the impact of the MOF on AlH_3_. The high‐resolution Al 2*p* XPS spectra (Figure 3f) show that the binding energy of Al^3+^ in the composite is positively shifted by 0.2 eV compared to the ball‐milled AlH_3_. The Al 2*p* spectrum of the composite sample shows a reduction in the Al^3+^ valence state and the formation of Al^0^ (ratio of 4.46%, Table S1, Supporting Information). This is attributed to the partial breakage of the Al─H bonds during ball‐milling, which is synergistically induced by mechanical forces and the catalytic sites of the MOF. Crucially, while the MOF exhibits a single Ti^4+^ state, the Ti in the AlH_3_‐MOF shows a mixed valence state of Ti^4+^ and Ti^3+^ (Figure 3g; Table S2, Supporting Information). These results indicate that the chemical binding of Al^3+^ on the MOF surface mainly occurs at Ti^4+^ sites, where directional electron sharing depletes the Al^3+^ electron density and activates the Al─H bonds for dehydrogenation. Simultaneously, Ti acts as an intermediate for electron transport during the photocatalytic dehydrogenation of AlH_3_.

### 2.3 Photocatalytic Dehydrogenation of AlH_3_‐MOF

As evidenced in Figure S6 (Supporting Information), AlH_3_‐MOF (1%) exhibits enhanced light absorption compared to α‐AlH_3_ in both the UV regions, particularly in the visible light regions. Based on spectral absorption characteristics, visible light was selected to induce the photocatalytic dehydrogenation of the AlH_3_‐MOF materials. Meanwhile, to assess application feasibility in abundant solar‐irradiated regions, a 25 °C circulating water‐cooling system was employed to simulate room‐temperature conditions, mitigating the ambient thermal fluctuations inherent to laboratory environments.

**Figure 4:**
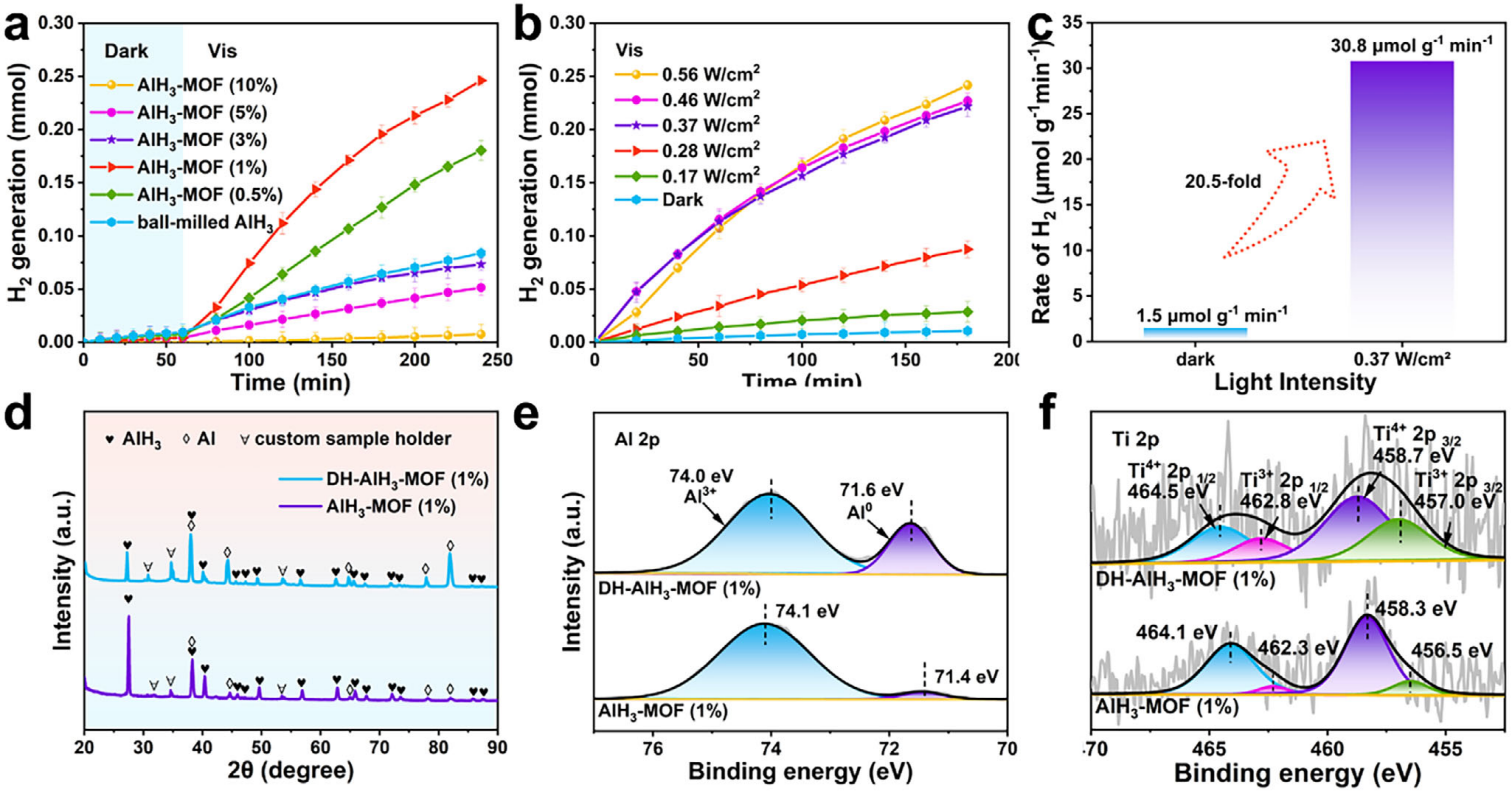
Visible‐light‐driven photocatalytic dehydrogenation performance and characterization of AlH_3_‐MOF. a) Visible‐light‐driven hydrogen release profiles of AlH_3_‐MOF and ball‐milled AlH_3_ under visible light irradiation (0.56 W cm^−2^) at room temperature (with error bars representing the standard deviation: *n* = 3). b) Visible‐light‐driven hydrogen release profiles of AlH_3_‐MOF (1%) under different light intensities at room temperature (with error bars representing the standard deviation: *n* = 3). c) Hydrogen release rates of AlH_3_‐MOF (1%) at room temperature under i) visible light irradiation (0.37 W cm^−2^) and ii) dark conditions. d) XRD patterns using a custom sample holder and the high‐resolution e) Al 2*p* and f) Ti 2*p* XPS spectra of DH‐AlH_3_‐MOF (1%) and AlH_3_‐MOF (1%).

A systematic investigation of the hydrogen desorption kinetics of AlH_3_‐MOF (x%, x = 0.5, 1, 3, 5, 10) and ball‐milled AlH_3_ was conducted under visible light irradiation (0.56 W cm^−2^) at room temperature (**Figures** **4**a; S7, Supporting Information). Among the composites, the AlH_3_‐MOF (1%) sample exhibits the best visible‐light‐driven photocatalytic dehydrogenation performance, releasing 3 times more hydrogen than that of ball‐milled AlH_3_. This illustrates that the MOF loading content should be controlled within a specific range to achieve the optimal photocatalytic performance. Notably, further increasing the MOF loading amount paradoxically diminishes the photocatalytic dehydrogenation performance. As evidenced in Figure S8 (Supporting Information), an excessive MOF loading induces photocatalyst agglomeration, which lowers the number of accessible active sites for AlH_3_ contact. Thus, the dehydrogenation kinetics are impeded. Furthermore, the thermally driven dehydrogenation performance of the composites (Figure S9, Supporting Information) confirms that incorporating a 1% MOF loading optimally enhances hydrogen desorption kinetics. The AlH_3_‐MOF (10%) composite exhibits a diminished thermally driven hydrogen capacity, which is attributed to the partial cleavage of Al─H bonds during ball‐milling (synergistically induced by mechanical force and the MOF catalytic sites).

The limitation of the 25 °C circulating water‐cooling system slows the photocatalytic dehydrogenation kinetics, extending the reaction times beyond practical measurement windows. Consequently, the visible‐light‐driven hydrogen release capacities (Figure S10, Supporting Information) were directly measured without active water‐cooling to obtain reliable data. Under visible‐light irradiation (0.56 W cm^−2^), 40 mg composite releases 0.7682–0.9519 mmol hydrogen. Next, the visible‐light‐driven hydrogen release capacities of AlH_3_‐MOF and ball‐milled AlH_3_ were compared with their thermally driven hydrogen release capacities in the absence of light (Table S2 and Figure S11, Supporting Information) to investigate the impact of visible light irradiation and external heating. The AlH_3_‐MOF (1%) sample has a visible‐light‐driven hydrogen release capacity of 4.7 wt.%, which is only 50% of that achieved under thermally driven conditions. This might be ascribed to the inherent limitation of simulated light irradiation penetrating the surface layer under the current experimental conditions, which would prevent complete dehydrogenation.

The light intensity is a key parameter influencing the visible‐light‐driven photocatalytic dehydrogenation performance of AlH_3_‐MOF. As displayed in Figure 4b,c, the influence of light intensity on the dehydrogenation performance of AlH_3_‐MOF (1%) was investigated at room temperature. Increasing the light intensity correspondingly increases the amount of hydrogen released from AlH_3_‐MOF (1%) until an irradiance threshold of 0.37 W cm^−2^ is reached, beyond which the hydrogen evolution rate stabilizes at 30.8 µmol g^−1^ min^−1^. This represented a greater than 20.5‐fold improvement compared to the performance of AlH_3_‐MOF (1%) under dark conditions. This irradiance threshold arises when the incident photon flux exceeds the maximum absorptive capacity of the MOF, saturating the yield of photogenerated charge carriers.^[^ ^38^ ^]^ The measured surface temperature of AlH_3_‐MOF (1%) under visible light irradiation (0.37 W cm^−2^) without active water‐cooling at a near‐ambient temperature is 55 °C (Figure S13, Supporting Information). In addition, isothermal dehydrogenation of this composite was performed at 55 °C. The maximum visible‐light‐driven hydrogen release capacity of this composite (4.7 wt.% within 180 min) is achieved at this near‐ambient temperature without external heating (Figure S14, Supporting Information). Meanwhile, a 2.1‐fold enhancement in the hydrogen release yield is achieved compared to conventional thermally driven dehydrogenation at a near‐ambient temperature for an identical duration. This illustrates the applicability of utilizing AlH_3_‐MOF for photocatalytic dehydrogenation under different conditions.

To further elucidate the underlying photocatalytic mechanism of AlH_3_‐MOF, the fundamental properties of AlH_3_‐MOF (1%) were characterized before and after dehydrogenation under visible light irradiation (0.37 W cm^−2^) at room temperature. The composite sample obtained after the dehydrogenation reaction is denoted DH‐AlH_3_‐MOF. XRD analysis (Figure 4d) reveals the visible‐light‐driven partial decomposition of AlH_3_ to Al NPs, although technical conditions constrained complete material decomposition. Correspondingly, digital images (Figure S15, Supporting Information) demonstrate a color transition from silvery‐white to dark‐gray, consistent with the partial conversion of AlH_3_ to Al NPs. SEM analyses (Figure S16, Supporting Information) indicate that the Al NPs obtained after dehydrogenation retain a particle size of 65 nm, which is identical to that of the AlH_3_ particles after mechanical ball‐milling. Furthermore, Al 2*p* XPS valence analyses (Figure 4e; Table S1, Supporting Information) reveal that the proportion of Al^0^ in the composite significantly increases to 23.74% after photocatalytic dehydrogenation, confirming the in situ formation of Al NPs during the dehydrogenation process. Concurrently, the Al^3+^ binding energy of 70.4 eV is negatively shifted by 0.1 eV, indicating a charge transfer‐driven reduction to Al^0^ through the acquisition of electrons during dehydrogenation. The high‐resolution Ti 2*p* XPS analyses (Figure 4f; Table S2, Supporting Information) demonstrate that the proportion of low‐valent Ti (Ti^3+^) increases to 37.52% after irradiation, while the Ti^4+^ binding energy exhibits an anomalously positive shift of 0.4 eV. This contradicts conventional expectations if Ti^4+^ only acquires electrons by bonding with the H^−^ of AlH_3_, the binding energy should decrease. This positive shift in the Ti^4+^ binding energy can potentially be attributed to the in situ generated Al NPs coordinating with the O atoms in Ti─O motifs, leading to a withdrawal of electrons from Ti^4+^ sites and a corresponding reduction in the local electron density. This analysis conclusively demonstrates the in situ formation of an Al/MOF heterostructure during visible‐light‐driven photocatalytic dehydrogenation. To establish quantitative correlations between Al content and photocatalytic dehydrogenation performance, a series of Al‐AlH_3_‐MOF composites with precisely controlled Al amount were synthesized via mechanical ball‐milling. Figure S17 (Supporting Information) demonstrates a positive correlation between Al content and dehydrogenation activity at a lower addition level. However, exceeding the optimal threshold paradoxically diminishes photocatalytic efficiency. These experiments establish a clear non‐monotonic relationship between Al content and catalytic performance.

### 2.4 Photocatalytic Dehydrogenation Mechanism of AlH_3_‐MOF

To unveil the impact of the in situ formation of the Al/MOF heterostructure on the visible‐light‐driven photocatalytic dehydrogenation kinetics, pristine Al/MOF was synthesized by mechanical ball‐milling. The resulting Al/MOF maintains the morphological and crystallographic integrity of the MOF matrix (Figure S19a, Supporting Information), confirming that the loading procedure preserves the intrinsic crystal phase of the MOF. The measured lattice spacing of 0.231 nm corresponds to the (111) plane of Al (Figure S19b, Supporting Information), directly confirming the incorporation of Al NPs. Complementary TEM‐EDS elemental mapping images (Figure S20, Supporting Information) indicate the uniform dispersion of the Al NPs across the MOF matrix in the composite photocatalyst. High‐resolution XPS analysis (Figure S21, Supporting Information) demonstrates the positive binding energy shifts of the Ti^4+^ and Ti─O components in the Ti 2*p* and O 1*s* spectra, confirming the synthesis of Al/MOF. This directional displacement indicates Al─O─Ti formation, which reduces the electron density around Ti^4+^ sites through covalent bond‐induced charge redistribution.

**Figure 5:**
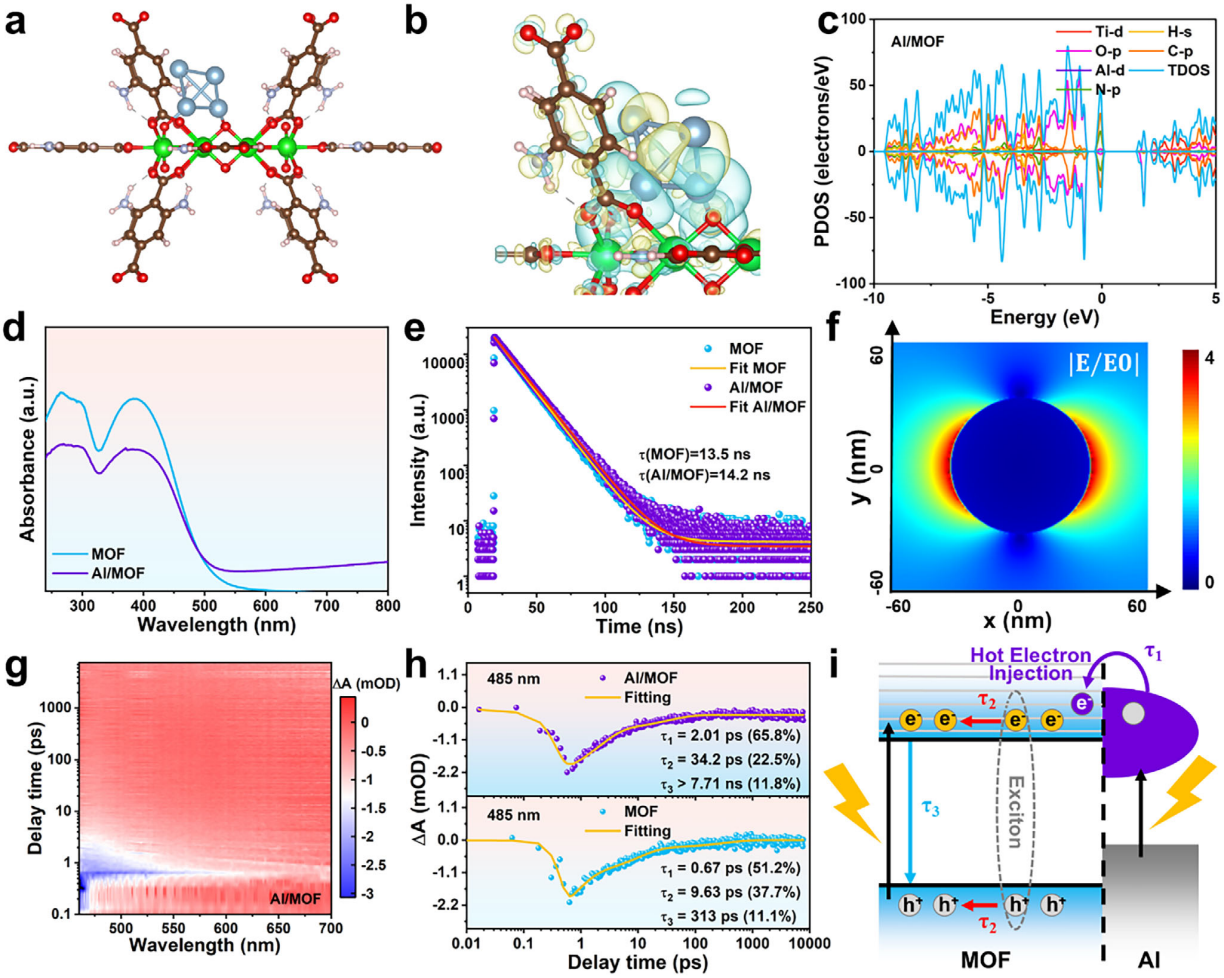
Photocatalytic mechanism studies of Al/MOF photocatalyst. a) Side view of optimized Al/MOF crystal mode. b) Side view of the electron density difference maps and c) DOS of Al/MOF. d) UV–vis absorption spectra and e) TRPL decay profiles of MOF and Al/MOF. f) Simulated interfacial electric field |E/E_0_| distribution of Al/MOF at 400 nm excitation wavelength. g) Fs‐TA contour maps of Al/MOF. h) Fs‐TA kinetics of MOF and Al/MOF probed at 485 nm. i) Schematic illustration of the visible‐light‐driven photocatalytic mechanism of Al/MOF.

DFT calculations were performed to identify the possible photocatalytic mechanisms for the dehydrogenation of AlH_3_ in Al/MOF under visible light irradiation. The structural model of Al/MOF was constructed according to the physical characterization results and structural optimization (**Figures** **5**a; S22, Supporting Information). As illustrated in Figures 5b and S23 (Supporting Information), the charge density distribution of this model indicates that interfacial electron transfer occurs from the Al NPs to the MOF matrix, as quantified by a 0.60 |e| Bader charge shift. Band structure analyses (Figure S24, Supporting Information) reveal that Al/MOF has a direct bandgap of 1.6 eV, signifying a 0.5 eV reduction compared to the pure MOF. This narrower bandgap extends the light absorption range of the composite, leading to enhanced photon‐to‐chemical energy conversion efficiency for AlH_3_ dehydrogenation. Moreover, DOS analysis (Figure 5c) reveals significant contributions from the Ti 3*d*, C 2*p*, O 2*p*, N 2*p*, H 1*s*, and Al 3*d* orbitals in Al/MOF. Crucially, the O 2*p* and C 2*p* orbitals dominate the highest occupied molecular orbital (HOMO), while the Ti 3*d* and C 2*p* orbitals govern the lowest unoccupied molecular orbital (LUMO), enabling optimized charge separation.

In Al/MOF, the MOF provides UV–vis absorption capabilities due to its narrow bandgap, while the Al NPs enable broadband LSPR spanning the UV to near‐infrared region. While the characteristic absorption peak intensity of Al/MOF diminishes, the synergy of these two components extends the visible light adsorption range of this composite, with the bandgap reduced from 2.64 to 2.52 eV (Figures 5d; S25, Supporting Information). Consequently, the visible light utilization efficiency is enhanced. Photoluminescence (PL) spectroscopy and photoelectrochemical measurements are frequently used to study the transfer and recombination kinetics of photogenerated charge carriers. Comparing the steady‐state PL spectra of the MOF and Al/MOF samples (Figure S26, Supporting Information) indicates that loading Al NPs leads to enhanced fluorescence. Generally, the PL intensity is the result of the radiative recombination of electron–hole pairs, meaning that a lower PL intensity indicates higher electron–hole pair separation efficiency.^[^ ^39^ ^]^ However, the time‐resolved photoluminescence (TRPL) decay profiles of these two samples (Figure 5e) indicate that photogenerated carriers have an extended lifetime in Al/MOF (***τ_1_*** = 14.2 ns) compared to the MOF sample (***τ_2_*** = 13.5 ns). Additionally, the MOF sample exhibits a photoluminescence quantum yield (PLQY) of 4.51%, while Al/MOF achieves an increased PLQY of 9.46%. The prolonged carrier lifetime and enhanced PLQY of Al/MOF demonstrate the more efficient photogenerated charge separation and transport properties of this composite. Meanwhile, the intensified steady‐state PL spectrum of the composite could be primarily attributed to the LSPR of the Al NPs, which amplifies the local electromagnetic field intensity around the MOF.^[^ ^27^ ^]^ This enhanced field promotes the generation of additional charge carriers within the MOF, resulting in an enhanced radiative recombination rate. Transient photocurrent response and electrochemical impedance spectroscopy (EIS) analyses were performed to elucidate the interfacial separation dynamics of photogenerated carriers in the composite photocatalyst. Across sequential on‐off photoirradiation cycles, the transient photocurrent response of Al/MOF demonstrates progressively enhanced charge separation (Figure S27a, Supporting Information). Complementary EIS Nyquist plots (Figure S27b, Supporting Information) reveal that the semicircle diameter of Al/MOF is smaller than that of MOF. Overall, the Al/MOF heterostructure exhibits improved photogenerated charge separation properties compared to the MOF structure. Mott‐Schottky analyses (Figure S28, Supporting Information) quantify an E_CBM_ shift from −0.62 V (MOF) to −0.77 V (Al/MOF) vs NHE, confirming a minor but consequential band energy reconfiguration upon the in situ formation of integrated plasmonic Al NPs. Synthesizing these findings, a mechanistic model involving band alignment and interfacial charge transfer pathways in AlH_3_‐Al/MOF is established (Figure S29, Supporting Information).

The LSPR excitation of the plasmonic metal causes the electrons of the plasmonic metal to gain energy exceeding the Fermi level, generating “hot electrons”. Previous studies have established that the generation rate of plasmonic decay‐driven hot electrons scales proportionally with the plasmon‐enhanced electromagnetic fields (|E/E_0_|).^[^ ^40^, ^41^ ^]^ To rigorously quantify the plasmonic hot electron dynamics in Al/MOF, a 3D finite‐difference time‐domain (FDTD) model (one Al NP with a diameter of 65 nm) integrating local electromagnetic field enhancement distributions was developed. As shown in Figure 5f, the enhanced local electromagnetic field near the edge of the plasmonic Al NPs on the Al/MOF interface is clearly observed upon 400 nm excitation, indicating the generation of hot electrons. Thus, the hot electron yield from non‐radiative plasmon decay is governed by the Al NPs‐induced enhancement of the local electromagnetic field |E/E_0_|. These hot electrons subsequently transfer to the conduction band (CB) of the MOF, where the semiconductor‐mediated suppression of electron‐electron scattering extends their lifetime.

The dynamics of the photophysical responses were explored via femtosecond transient absorption (fs‐TA) spectroscopy to compare the carrier relaxation pathways in the MOF and Al/MOF materials upon photoexcitation. The samples were excited by a 400 nm laser pulse and probed with a white light continuum. As demonstrated by the fs‐TA contour maps (Figures 5g; S30, Supporting Information) and spectra acquired at selected pump‐probe delays (Figure S31, Supporting Information), both samples exhibit superimposed ground state bleaching (GSB) and simulated emission (SE) signals centered ≈485 nm.^[^ ^42^ ^]^ The results indicate that both systems have analogous band‐edge excitation features across the visible region. As shown in Figure 5h, the triple‐exponential decay fitting of the kinetic traces of Al/MOF at 485 nm reveals three lifetimes: ***τ_1_*** = 2.01 ps (65.8%), ***τ_2_*** = 34.2 ps (22.5%), and ***τ_3_*** >7.71 ns (11.8%, beyond the instrument window). A comparison with the MOF lifetimes (***τ_1_*** = 0.67 ps (51.2%), ***τ_2_*** = 9.63 ps (37.7%), and ***τ_3_*** = 313 ps (11.1%)) confirms that these two samples have analogous carrier relaxation pathways but critical divergences. The ***τ_1_*** lifetime is mainly attributed to hot carrier cooling (HCC) and defect/surface trapping in the MOF. The prolonged ***τ_1_*** lifetime and augmented amplitude in Al/MOF indicate that hot electron injection from the plasmon Al NPs becomes the dominant contribution, partially offsetting HCC/trapping recovery.^[^ ^43^ ^]^ The extended ***τ_2_*** lifetime of Al/MOF arises from the LSPR‐induced enhancement of the interfacial electromagnetic field accelerating exciton dissociation into free carriers. The ***τ_3_*** lifetime is primarily governed by free carrier recombination processes. Notably, the ***τ_3_*** lifetime of Al/MOF is prolonged to >7.71 ns, and an enhanced amplitude is also achieved. This enhancement is attributed to the injection of hot electrons, which facilitates the spatial separation of free carriers. Meanwhile, the localized electromagnetic field generated by LSPR not only accelerates exciton dissociation into free carriers but also imparts a greater initial separation distance to the charge carriers, thereby suppressing their recombination.^[^ ^44^ ^]^ Clearly, the plasmon‐injected hot electrons from the Al NPs effectively compensate for carrier relaxation losses in the MOF, resulting in extended effective carrier lifetimes and enhanced charge separation, which boosts the photocatalytic activity of Al/MOF^[^ ^45^ ^]^ for AlH_3_ dehydrogenation.

**Figure 6:**
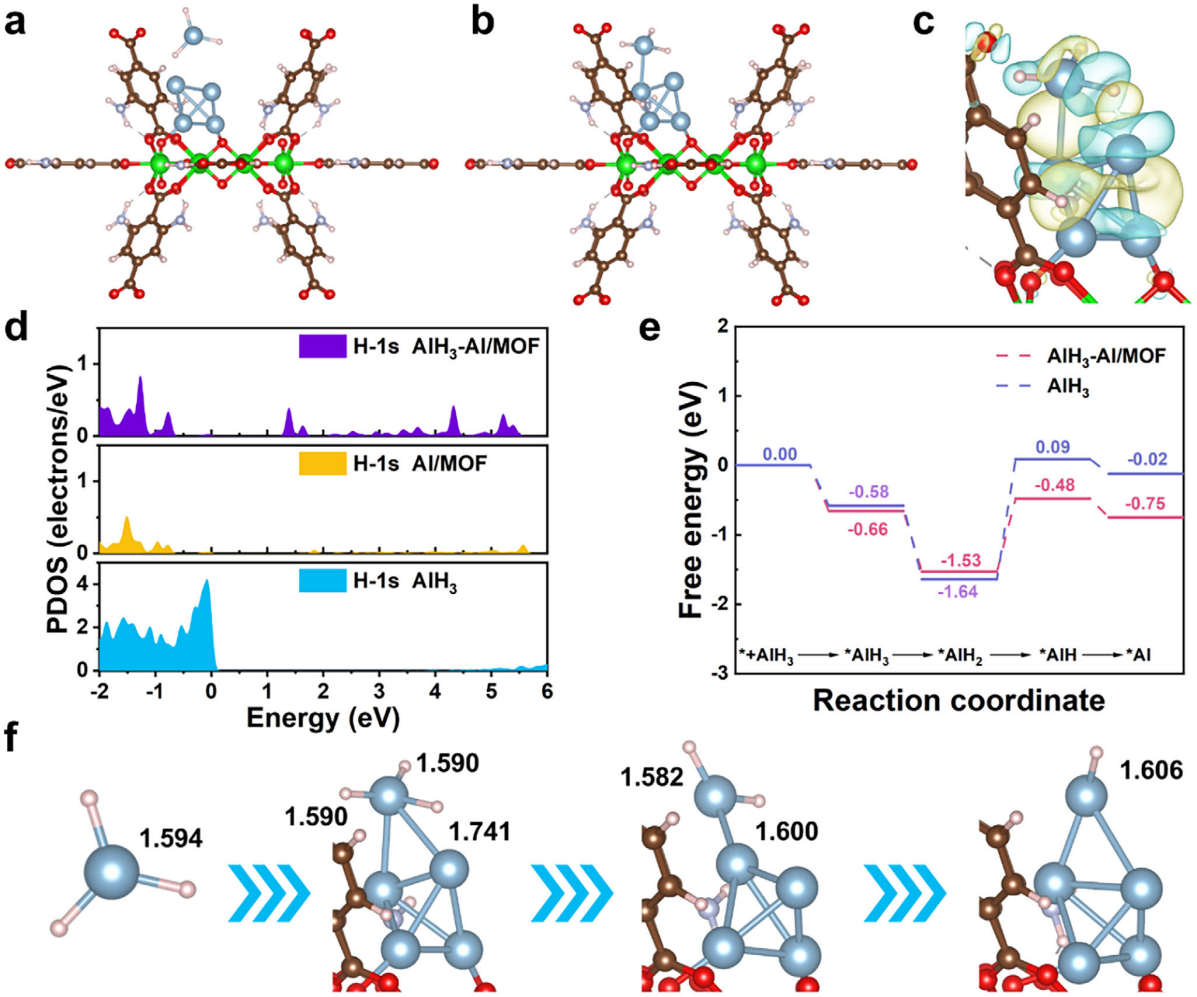
Density functional theory calculations of AlH_3_‐Al/MOF. Side view of optimized structures a) before and b) after adsorption of AlH_3_ cluster on Al/MOF surface. c) Side view of the electron density difference map of AlH_3_‐Al/MOF. d) H‐1*s* PDOS of AlH_3_, Al/MOF, and AlH_3_‐Al/MOF. e) Free energy profiles for AlH_3_ complexation and stepwise dehydrogenation on Al/MOF surface. f) Al─H bond lengths of AlH_3_ after adsorption on Al/MOF surface.

DFT calculations were performed to explain the photon‐driven charge transfer pathways and rate‐determining Al─H bond cleavage steps. **Figure** **6**a,b displays the optimized structures before and after the adsorption of an AlH_3_ cluster on Al/MOF. Additionally, charge transfer analyses (Figures 6c; S32, Supporting Information) were employed to better understand the redistribution of electrons in AlH_3_‐Al/MOF. The electron density difference of AlH_3_‐Al/MOF suggests that electrons are transferred from the Al/MOF surface to the AlH_3_ cluster, weakening the Al─H bonds.^[^ ^46^ ^]^ According to the DOS calculations (Figure 6d), the increased H‐1*s* partial density of states (PDOS) form 0–5 eV confirms orbital hybridization between Al/MOF and the Al─H bonds, which generates new hybridized orbitals. This hybridization significantly activates and weakens the Al─H bonds and also localizes new orbitals near the Fermi level—precisely within the active energy window for the migration of photogenerated carriers. Consequently, an efficient interfacial charge transfer channel is provided to drive Al─H bond cleavage. These results indicate that the adsorption of AlH_3_ on Al/MOF leads to hybridization and electron redistribution, which synergistically weakens the Al─H bonds, establishing a direct interfacial charge transfer channel to drive Al─H bond cleavage.

The free energy profiles of AlH_3_ complexation and stepwise dehydrogenation on the Al/MOF surface were computed from the optimized structures (Figure 6e). This analysis reveals that AlH_3_ complexation on the Al/MOF surface is accompanied by a free energy change of −0.66 eV, confirming spontaneous adsorption. The pure AlH_3_ dehydrogenation pathway exhibits an overall endergonic shift of +0.56 eV (ΔG >0), which renders this pathway thermodynamically unfavorable. Remarkably, coupling AlH_3_ with the photocatalyst drives an identical dehydrogenation pathway to an exergonic state (ΔG = −0.09 eV), enabling a spontaneous reaction. Crucially, photoinduction reduces the rate‐determining barrier (^*^AlH_2_ → ^*^AlH) by 39% (from 1.73 to 1.05 eV) via selective Al─H σ^*^ antibonding orbital population by photogenerated carriers. The Al─H bond lengths were determined using the optimized structure after AlH_3_ cluster adsorption on the Al/MOF surface, as plotted in Figure 6f. The adsorption of AlH_3_ on the photocatalyst surface disrupts the molecular symmetry, resulting in two shortened Al─H bonds (1.590 Å) and one elongated Al─H bond (1.741 Å). This selective bond activation indicates that the elongated bond is the preferential cleavage site. The resulting AlH_2_ intermediate exhibits further asymmetry, with the bond lengths diverging to 1.582 and 1.600 Å. This facilitates a directed second dehydrogenation step. During the rate‐determining step (^*^AlH_2_ → ^*^AlH), photoinduced holes populate the σ^*^ orbital of the elongated bond (1.606 Å). This process demonstrates a stepwise bond‐polarization mechanism, which is fundamentally driven by photogenerated carriers mediating a dynamic electron redistribution toward the Al─H bonds.

## 3 Conclusion

In summary, this study demonstrates the in situ construction of an Al/MOF photocatalyst during the visible‐light‐driven photocatalytic dehydrogenation of AlH_3_‐MOF. This process achieves the non‐thermal modulation of the dehydrogenation reaction via photogenerated carrier‐directed AlH_3_ interactions. The plasmonic Al NPs in Al/MOF expand the light‐harvesting range and enhance the charge separation efficiency through hot electron injection. An FDTD simulation and fs‐TA measurements collectively verify the transfer of hot electrons from the Al NPs to the MOF, providing a supply of additional carriers to drive the photocatalytic dehydrogenation of AlH_3_. Moreover, DFT calculations reveal that the chemisorption of AlH_3_ at the Al/MOF interface induces interfacial electron redistribution and enhances the interfacial charge transfer channel, expediting Al─H bond breaking and the subsequent release of hydrogen. Experimental results demonstrate that AlH_3_‐MOF (1%) achieves a hydrogen release rate of 30.8 µmol g^−1^ min^−1^ under visible‐light irradiation at room temperature, representing a 20‐fold enhancement compared to dark conditions. Additionally, AlH_3_‐MOF (1%) releases 4.7 wt.% hydrogen within 180 min at a near‐ambient temperature under an ultra‐low light intensity of 0.37 W cm^−2^ without external heating. This study highlights the visible‐light‐driven photocatalytic performance of the Al/MOF heterostructure for the dehydrogenation of high‐density hydrides, emphasizing a photocatalytic mechanism rather than a photothermal mechanism. The non‐thermodynamic photocatalytic regulation pathway pioneered in this work offers a new paradigm for designing highly efficient solar conversion hydrogen supply systems based on solid‐state high‐energy hydrides. However, realizing reversible storage necessitates future breakthroughs in the photocatalytic hydrogenation of AlH_3_ under mild conditions, a critical frontier for achieving solar‐to‐hydrogen energy cycles.

## 4 Experimental Section

### Materials

2‐aminoterephthalic acid (NH_2_‐BDC) was purchased from Thermo Scientific. N,N‐Dimethylformamide (DMF) was acquired from Adamas‐beta. Methanol (MeOH) and titanium (IV) isopropoxide (TTIP) were obtained from Sigma–Aldrich. α‐AlH_3_ was purchased from Henan Nayu New Material Co., Ltd. Tetrahydrofuran (THF) was acquired from Energy Chemical. All chemicals were used without further purification.

### Synthesis of NH_2_‐MIL‐125

NH_2_‐MIL‐125 was synthesized by a solvothermal method. NH_2_‐BDC (2.86 g) was dissolved in a mixed solution comprising DMF (40 mL) and MeOH (10 mL), which was stirred until complete dissolution was achieved. Subsequently, TTIP (2.86 mL) was added to the mixed solution, followed by sonication for 15 min. The solution was transferred into a 100 mL Teflon‐lined stainless‐steel autoclave and heated at 110 °C for 72 h. After cooling to room temperature, the resulting solid material was washed with DMF and MeOH and dried in a vacuum oven at 60 °C for 24 h.

### Synthesis of AlH_3_‐MOF

The AlH_3_‐MOF (x%) composites (x refers to the mass doping ratio of MOF in the composite) were synthesized via mechanical ball‐milling. A mixture of α‐AlH_3_ and MOF (NH_2_‐MIL‐125, x wt.%) was added to a 100 mL zirconia container under an argon atmosphere. The ball‐to‐powder weight ratio was controlled at 30:1, and the rotation speed of the miller was maintained at 450 rpm (with alternating clockwise and counterclockwise rotations). A 5 min pause was implemented after every 5 min ball‐milling step. Under these conditions, AlH_3_‐MOF (x%) (x = 0, 0.5, 1, 3, 5, 10) was mechanically milled for 1 h. All samples were handled in an argon‐filled glove box (O_2_ and H_2_O <0.1 ppm). Ball‐milled AlH_3_ was similarly synthesized.

### Synthesis of Al‐AlH_3_‐MOF

The Al‐AlH_3_‐MOF composites were synthesized by mechanical ball‐milling. Highly active Al powder was obtained through the dehydrogenation of α‐AlH_3_. A mixture consisting of highly active Al powder (x%), MOF (1%), and AlH_3_ was added to a 100 mL zirconia container under an argon atmosphere. The ball‐to‐powder weight ratio was controlled at 30:1, and the rotation speed of the miller was maintained at 450 rpm (with alternating clockwise and counterclockwise rotations). A 5 min pause was implemented after every 5 min ball‐milling step. Under these conditions, the Al‐AlH_3_‐MOF composites were mechanically milled for 1 h. The samples were handled in an argon‐filled glovebox (O_2_ and H_2_O <0.1 ppm).

### Synthesis of Al/MOF

The Al/MOF photocatalyst was synthesized by mechanical ball‐milling. Highly active Al NP powder was obtained via the dehydrogenation of α‐AlH_3_, and the Al NP content of the composite photocatalyst was 1 wt.%. A mixture of the highly active Al NPs and the MOF was added to a 100 mL zirconia container under an argon atmosphere. The ball‐to‐powder weight ratio was controlled at 30:1, and the rotation speed of the miller was maintained at 450 rpm (with alternating clockwise and counterclockwise rotations). A 5 min pause was implemented after every 5 min ball‐milling step. Under these conditions, the Al/MOF photocatalyst was mechanically milled for 1 h. The sample was handled in an argon‐filled glove box (O_2_ and H_2_O <0.1 ppm).

### Materials Characterization

The morphologies of the composite materials were observed by scanning electron microscopy (SEM, ZEISS, Sigma 500) and transmission electron microscopy (TEM, JEOL JEM‐2100F). X‐ray diffraction (XRD, D8 Advance, Bruker AXS) was used to determine the phase compositions. A custom XRD sample holder was employed to protect materials from oxidation using by Kapton tape, which is amorphous and exhibits a broad peak ≈2θ ≈10 ≈20°. All composites containing α‐AlH_3_ and Al NPs were prepared in an argon glove box and then covered with Kapton tape prior to XRD measurements. FT‐IR spectroscopy was performed using a Thermo Scientific Nicolet iS50 FTIR spectrometer operated at room temperature. A custom sample holder with KBr windows was utilized to prevent oxidative degradation during the FT‐IR testing. X‐ray photoelectron spectroscopy (XPS, Thermo Scientific EscaLab 250Xi) was employed to investigate the surface composition and elemental valence states. The Brunauer–Emmett–Teller (BET) surface area and pore structure were measured by obtaining N_2_ adsorption‐desorption isotherms using a self‐adsorption‐intelligence‐MP‐C device. UV–vis diffuse reflectance spectroscopy was performed using a UV–vis spectrophotometer (UV‐2600i, Shimadzu). Steady‐state photoluminescence (PL) spectra were recorded on an F‐4700 fluorescence spectrometer under 355 nm excitation. A 300 W Xe lamp (PLS‐SXE300+, Beijing Perfectlight) was used as the light source, and an optical power meter (PL‐MW2000, Beijing Perfectlight) was adopted to measure the light intensity.

### Femtosecond Transient Absorption (fs‐TA) Measurements

Femtosecond‐transient absorption measurements were conducted using a Helios pump‐probe system (Ultrafast Systems) in collaboration with a regenerative amplified laser system (Spectra‐Physics, Solstice Ace). A Ti: sapphire amplifier was used to generate an 800 nm pulse with a repetition rate of 1 kHz, a duration of <120 fs, and an energy of 7 mJ  per pulse. The output of the amplifier was split into two beams. One beam was used to pump an optical parametric amplifier (Ultrafast Systems, APOLLO‐T), while the other beam was focused onto a sapphire crystal to generate a white light supercontinuum (from 430 to 780 nm) as the probe beam. The delay time between the pump and probe pulse was varied by a smart optical delay line (minimum step size of 2.8 fs, bi‐directional repeatability of 14 fs). Before the test, samples were evenly dispersed in THF in an argon glove box (O_2_ and H_2_O <0.1 ppm). Each dispersion was sealed in a quartz tube in the glovebox prior to the fs‐TA tests. The wavelength of the pump laser was set at 400 nm, and the pump fluence was 0.72 µJ cm^−2^.

### Photoelectrochemical Measurements

Photocurrent measurements and Mott‐Schottky plot measurements were performed in a standard three‐electrode system on a CHI650E electrochemical workstation (Chenhua Instrument, Shanghai, China). Typically, 2 mg sample was dispersed in a solution consisting of 1 mL ethanol and 10 µL of 5 wt.% Nafion. The working electrode was prepared by coating 200 µL of this mixed solution on the surface of an indium tin oxide (ITO) plate with an area of 4 cm^2^. A Pt plate and an Ag/AgCl electrode were used as the counter electrode and reference electrode, respectively. A 0.5 m Na_2_SO_4_ solution was prepared for use as the electrolyte. Visible‐light irradiation was used as the light source. The photocurrent responsive signals of the samples were measured under chopped light with a 0.5 V bias potential. Frequencies of 500, 1000, and 1500 Hz were employed for Mott‐Schottky analysis.

Electrochemical impedance spectroscopy (EIS) was performed in a standard three‐electrode system on a CHI650E electrochemical workstation (Chenhua Instrument, Shanghai, China). Typically, 2 mg photocatalyst was dispersed in a mixed solution consisting of 1 mL of ethanol and 10 µL of 5 wt.% Nafion. A glassy carbon substrate (Φ = 3 cm) coated with 20 µL mixed solution was used as the working electrode. A Pt plate was used as the counter electrode, and an Ag/AgCl electrode was used as the reference electrode. A 0.5 m Na_2_SO_4_ solution was prepared for use as the electrolyte. EIS was performed with a bias potential of −1.5 V in the dark.

### Calculation of Photoluminescence Quantum Yield (PLQY)

The photoluminescence quantum yield (PLQY) was calculated according to integrating sphere spectrometry. By comparing the emission spectra from the sample‐loaded cavity (*E*_c_: sample emission, *L*_c_: sample scattering) and the empty reference cavity (*E*_a_: reference emission, *L*_a_: reference scattering), the PLQY was calculated as shown in Equation (1).

(1) $$\emptyset_{f}=\frac{E_{c}\;-\;\left(1-A\right)\cdot E_{b}}{L_{a}\cdot A}=\frac{E_{c}\;-\;E_{a}}{L_{a}\;-\;L_{c}}$$

where *E*_b_ represents the integrated fluorescence induced by indirect illumination from the sphere wall, and *A* represents the absorptance of the sample at the excitation wavelength.

### Dehydrogenation Measurements

The thermally driven dehydrogenation performance of AlH_3_‐MOF and ball‐milled AlH_3_ was studied using temperature‐programmed desorption (TPD) at a heating rate of 2 °C min^−1^ and isothermal dehydrogenation tests with a high‐pressure pressure‐composition‐temperature (PCT) adsorption analyzer (H‐Sorb 2600PCT, CIQTEK Co., Ltd.).

Typically, the visible‐light‐driven dehydrogenation of AlH_3_‐MOF and ball‐milled AlH_3_ was carried out in an optical reaction vessel (≈150 mL), and a 25 °C circulating water‐cooling system was employed to simulate room temperature conditions. Within an argon‐filled glove box (O_2_ and H_2_O <0.1 ppm), a 40 mg sample was uniformly dispersed on a 7.07 cm^2^ Petri dish, which was then positioned inside the optical reaction vessel. The reaction vessel was hermetically sealed inside the glove box before extraction. The reaction vessel was then fixed and irradiated by a 300 W Xenon lamp (PLS‐SXE300+, Beijing Perfectlight) equipped with a UV‐cut filter to simulate visible light irradiation. The generation of hydrogen gas was measured by gas chromatography (GC, JieDao GC1690, argon as the carrier gas) using a thermal conductivity detector (TCD). Before the test, a calibration curve was obtained by using standard hydrogen with known concentrations, enabling the conversion of the GC peak area into the concentration of hydrogen. For each hydrogen generation evaluation test, 500 µL of the headspace was injected into the GC and quantified by a calibration plot to the internal hydrogen standard.

### Calculation of Hydrogen Release Capacity

The hydrogen release capacity of AlH_3_‐MOF was calculated according to the following equations:

(2) $$m\left(H_{2}\right)=n\left(H_{2}\right)\cdot M\left(H_{2}\right)$$

(3) $$\begin{array}{c} Hydrogen\;Release\;Capacity=\frac{m\left(H_{2}\right)}{m\left(composite\right)\;-m\left(MOF\right)}\;\times 100\%\;\left(wt.\%\right) \end{array}$$

where m(composite) represents the mass of AlH_3_‐MOF.

### FDTD Simulation

In this paper, FDTD was used to calculate the electric field distribution of the prepared nanostructures. In the calculation process, a perfect matching layer condition was applied in the x, y, and z directions to effectively prevent unphysical scattering. A mesh size of 0.25 nm × 0.25 nm × 0.25 nm was employed for the whole simulation area to obtain accurate calculation results. Importantly, the total‐field scattering field plane wave was adopted as the excitation light source incident vertically along the z‐direction to the surface of the nanostructures. The simulation convergence time was set to 1 × 10^−5^ fs to ensure the convergence of the computational results. Finally, an electric field monitor was employed to obtain the electric field distribution of the nanostructures.

### DFT Calculations

Spin‐polarized density functional theory (DFT) calculations^[^ ^47^, ^48^ ^]^ were carried out in the Vienna ab initio simulation package (VASP) based on the plane‐wave basis sets with the projector augmented‐wave method.^[^ ^49^, ^50^ ^]^ The exchange‐correlation potential was treated by using a generalized gradient approximation (GGA) with the Perdew–Burke–Ernzerhof (PBE) parametrization.^[^ ^51^ ^]^ The energy cutoff was set to 400 eV. The Brillouin‐zone integration was sampled with a Γ‐centered Monkhorst‐Pack mesh^[^ ^52^ ^]^ of 1 × 1 × 1 for the MOF, 9 × 9 × 2 for the AlH_3_ bulk, and 2 × 3 × 1 for the AlH_3_ surface by VASPKIT.^[^ ^53^ ^]^ The structures were fully relaxed until the maximum force on each atom was less than 0.02 eV Å^−1^, and the energy convergence standard was 10^−5^ eV. The van der Waals correction of Grimme's DFT‐D3 model was also adopted.^[^ ^54^ ^]^ To avoid periodic interactions between interface structures, a vacuum layer as large as 15 Å was used along the c direction normal to the interface.

For each subsequent elementary step, the Gibbs free energy was calculated after gas correction according to the following equation:

(4) ΔG=ΔE−ΔZPE+TΔS

where Δ*E* is the reaction energy obtained by determining the total energy difference between the reactant and product molecules absorbed on the catalyst surface, and Δ*S* is the change in entropy for each reaction. A room temperature of *T* = 298.15 K was used in the calculations to consider the influence of temperature. ΔZPE is the zero‐point energy correction for the Gibbs free energy.

### Statistical Analysis

All experimental data were processed and analyzed using standard statistical methods. Before analysis, raw data were examined for outliers and, where necessary, normalized to account for systematic variations. Data are presented as mean ± standard deviation (SD), derived from at least three independent experiments (*n* ≥3). All data processing was performed by the software Origin.

## Conflict of Interest

The authors declare no conflict of interest.

## Supporting information



Supporting Information

Supplemental Video 1

## Data Availability

The data that support the findings of the study are included in the main text and supporting information files.
