# Supplementary material for: Room‐Temperature Alane Dehydrogenation for Visible‐Light‐Driven Photocatalytic Hydrogen Supply System
Source: Adv Sci (Weinh). 2025 Dec 12;13(11):e18927. doi: 10.1002/advs.202518927 (PMC12931247; doi:10.1002/advs.202518927)
Supplement: Supplementary file 1 — Supporting Information [file ADVS-13-e18927-s001.doc]

**Supplementary Information**

**Room-temperature Alane Dehydrogenation for Visible-light-driven Photocatalytic Hydrogen Supply System**

Ting-Ting Li^1^, Li-Cheng Liang^1^, Rui-Qi Chen^2^, Chun-Qi Zhang^1^, Sheng-Nan Zhang^1^, Wen-Wen Cheng^1^, Xi-Hao Chen^3^, Ning Wang^4^, Juan-Ding Xiao*^1^, Qing-Qing Yang*^2^, Fei Liang*^1^ & Chao-Feng Zhang^1^

^1^Institutes of Physical Science and Information Technology, Anhui Graphene Carbon Fiber Materials Research Center, Anhui University, Hefei 230601, China.

^2^School of Materials Science and Engineering, Anhui Provincial Key Laboratory of Magnetic Functional Materials and Devices, Center for Free Electron Laser & High Magnetic Field, Anhui University, Hefei 230601, China.

^3^School of Materials Science and Engineering, Chongqing University of Arts and Sciences, Chongqing 402160, China.

^4^School of Science, Key Laboratory of High Performance Scientific Computation, Xihua University, Chengdu 610039, China.

E-mails: [jdxiao@ahu.edu.cn](mailto:jdxiao@ahu.edu.cn); [qqyang@ahu.edu.cn](mailto:qqyang@ahu.edu.cn); [fliang@ahu.edu.cn](mailto:fliang@ahu.edu.cn)

## Supplementary Figures


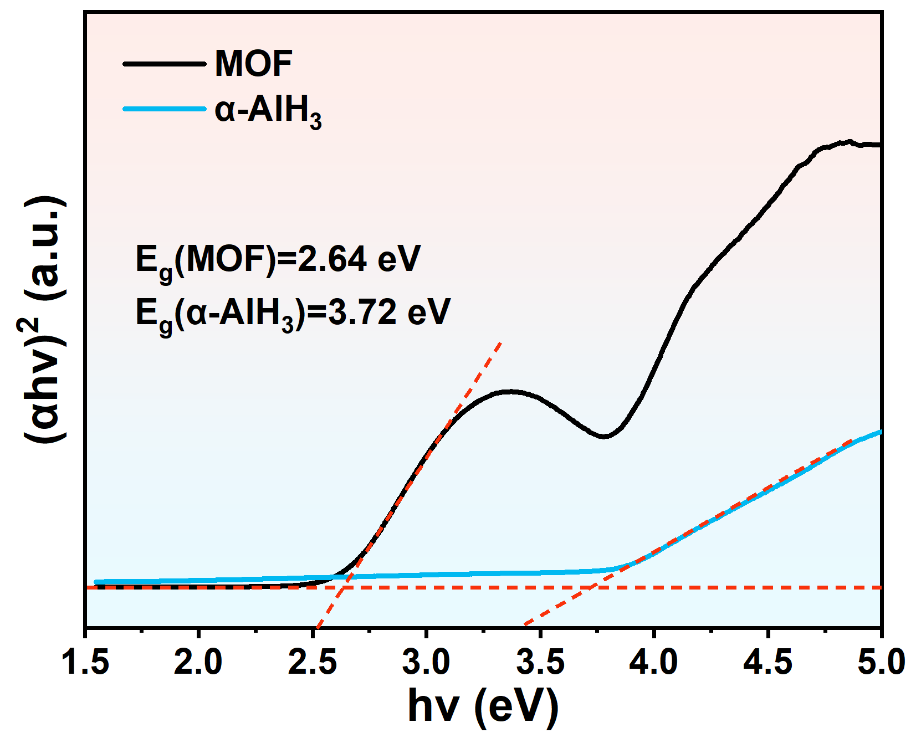


**Supplementary Figure 1.** Tauc plots for energy gap of MOF and ball-milled AlH_3_.


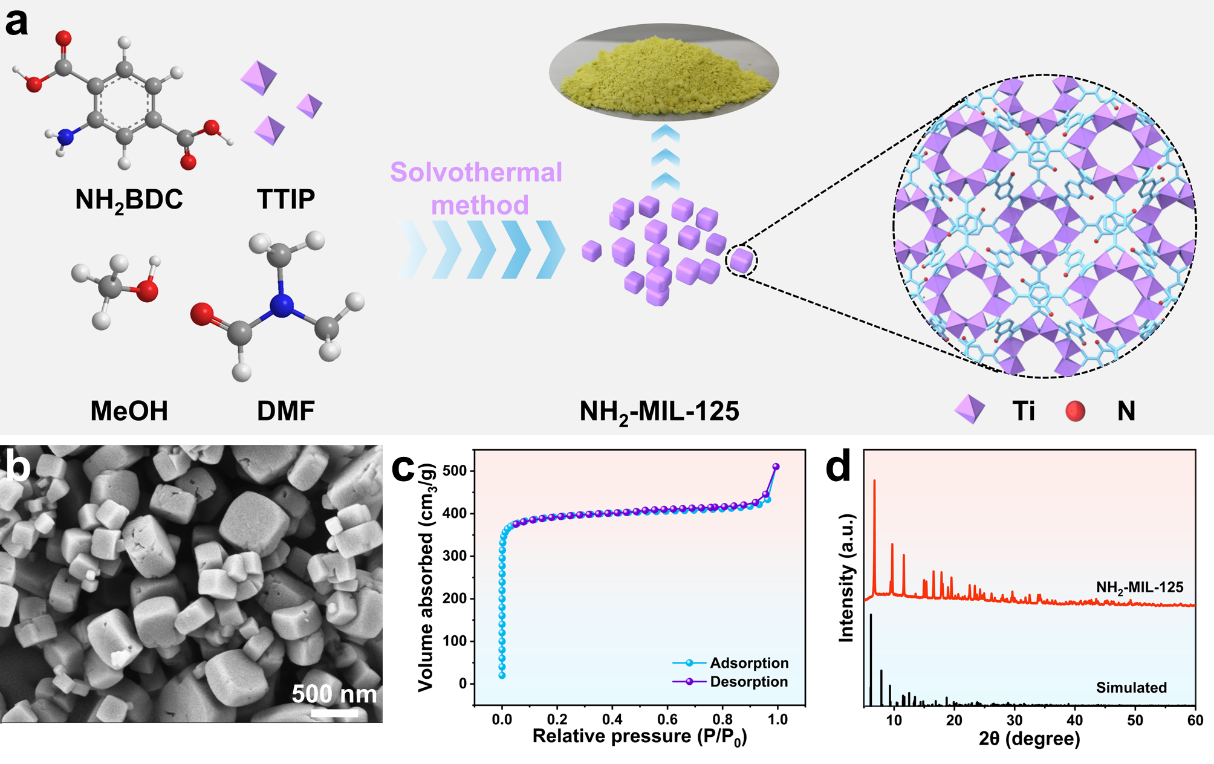


**Supplementary Figure 2.** **a** Schematic illustration of the fabrication of NH_2_-MIL-125. **b** SEM image, **c** BET and **d** conventional XRD pattern of the as-synthesized of NH_2_-MIL-125.


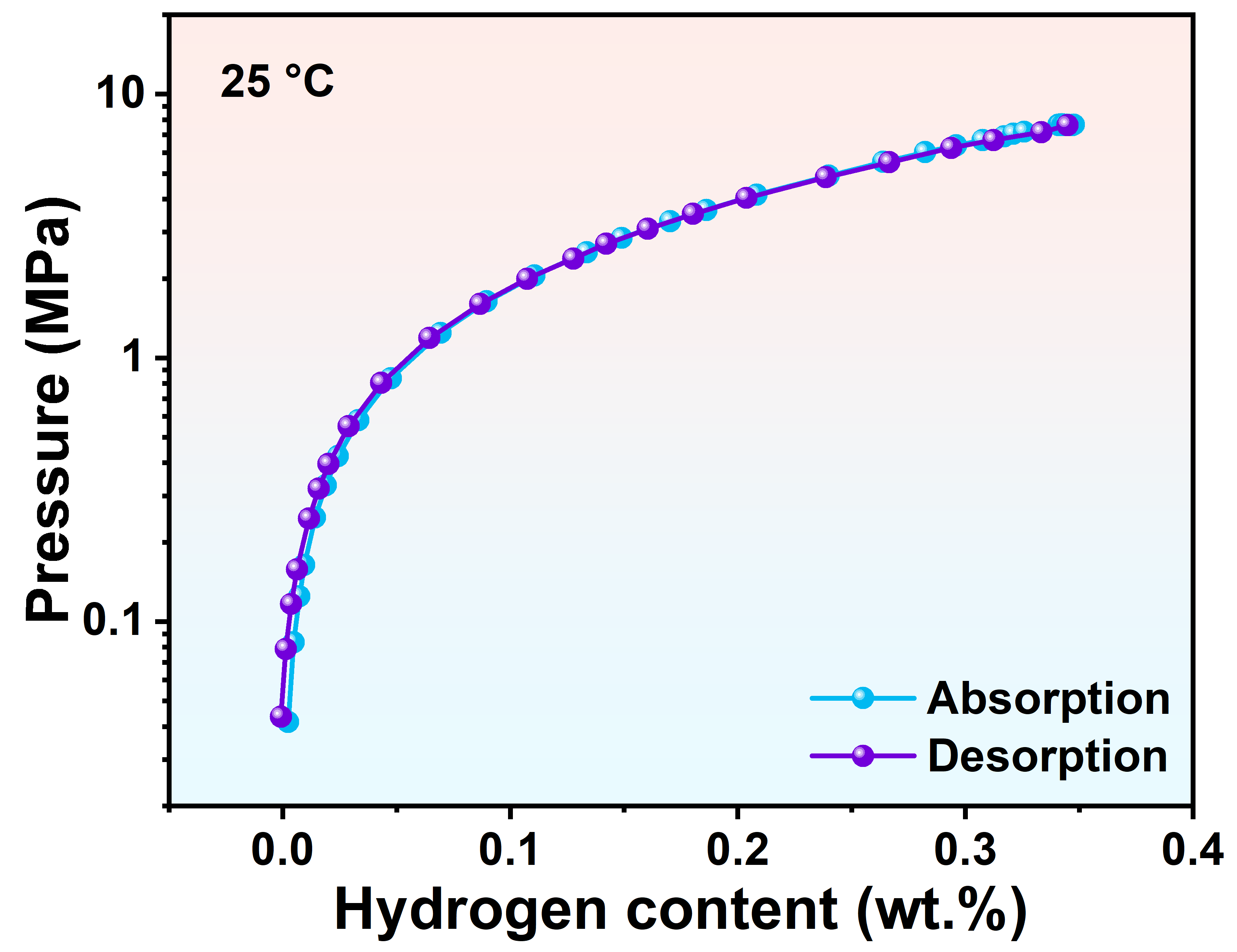


**Supplementary Figure 3.** Hydrogen adsorption-desorption isotherm measurement of MOF at 25°C。

The MOF exhibits a hydrogen sorption capacity of merely 0.056 wt.% at 1 MPa pressure and 25°C. More importantly, MOF hardly absorbs hydrogen at normal pressure (0.1 MPa) and 25°C. And as a photocatalyst, the content of MOF is extremely small and can be ignored. Although MOF are porous materials suitable for physical adsorption of hydrogen, the above content explains that MOF will not lead to a loss in the hydrogen capacity of AlH_3_-MOF during photocatalytic dehydrogenation.


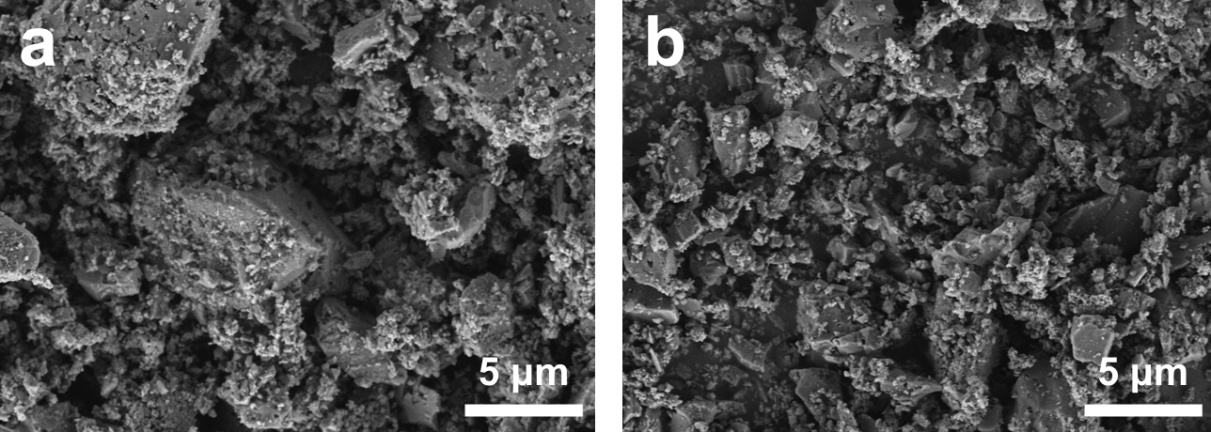


**Supplementary Figure 4.** SEM images of **a** ball-milled AlH_3_ and **b** AlH_3_-MOF (1%).


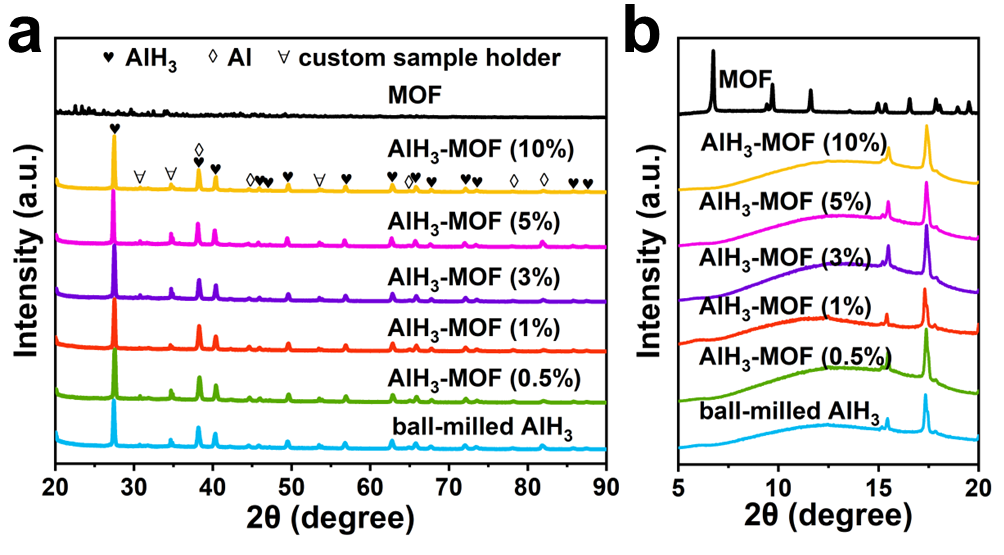


**Supplementary Figure 5.** XRD patterns using custom sample holder (Kapton tape-protected) of AlH_3_-MOF and ball-milled AlH_3_ at **a** 20-90° and **b** 5-20°.


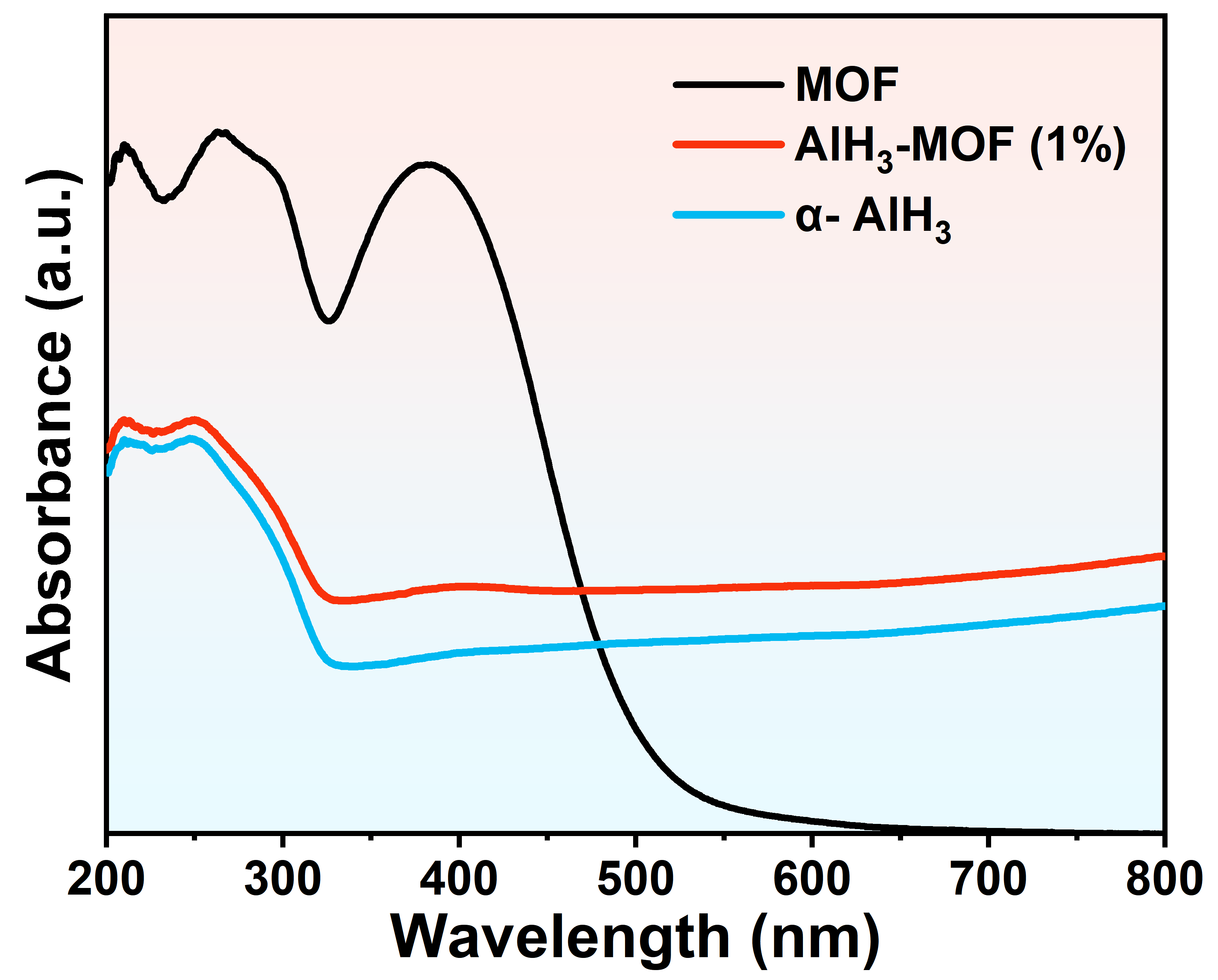


**Supplementary Figure 6.** UV-vis absorption spectroscopy of α-AlH_3_, MOF and AlH_3_-MOF (1%).


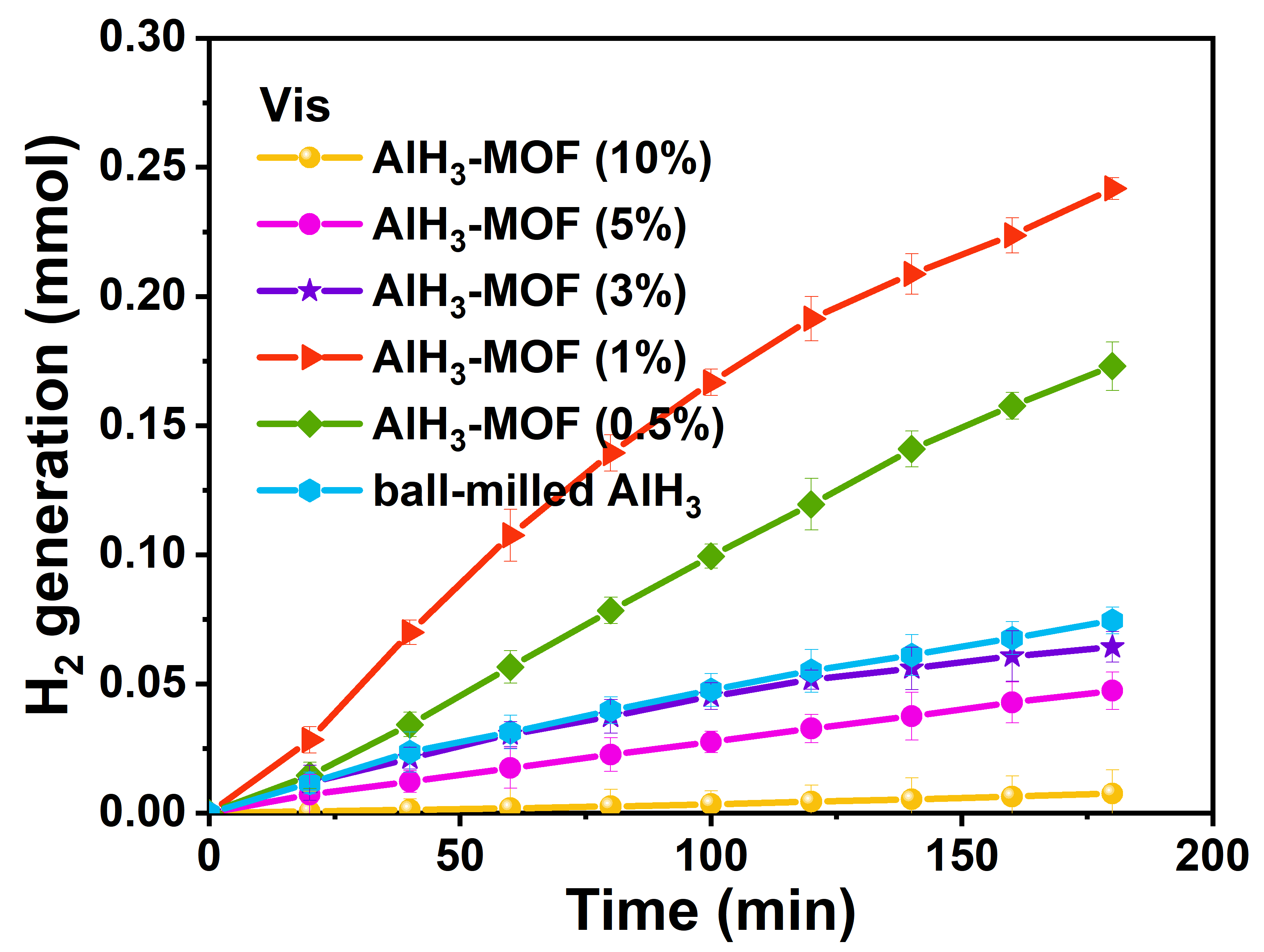


**Supplementary Figure 7.** Visible-light-driven hydrogen release profiles of AlH_3_-MOF and ball-milled AlH_3_ without dark conditions under visible light irradiation (0.56 W/cm^2^) and at room temperature (with error bars representing the standard deviation: n=3).


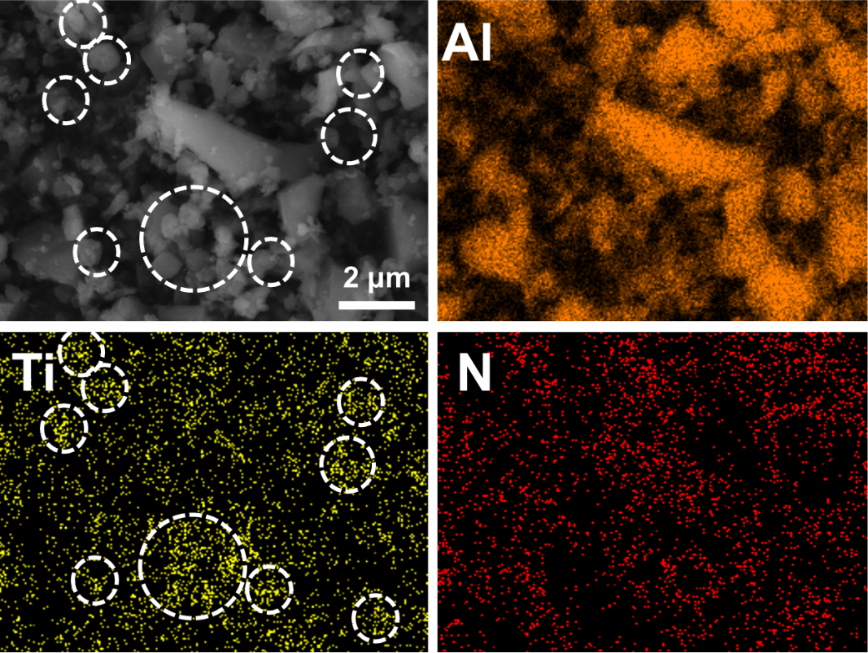


**Supplementary Figure 8.** TEM-EDS elemental mapping images of AlH_3_-MOF (10%).


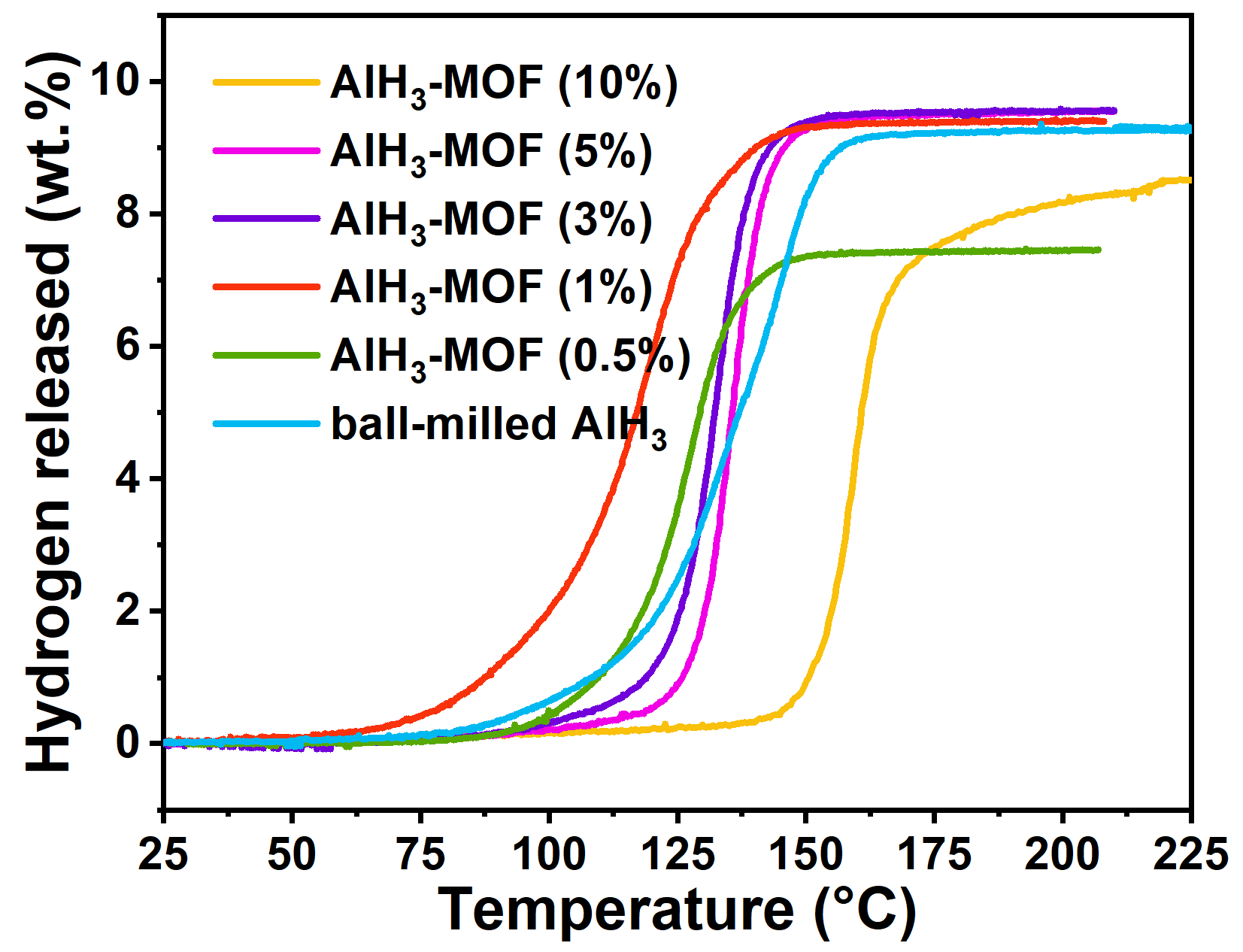


**Supplementary Figure 9.** Thermal-driven hydrogen release profiles of AlH_3_-MOF and ball-milled AlH_3_.


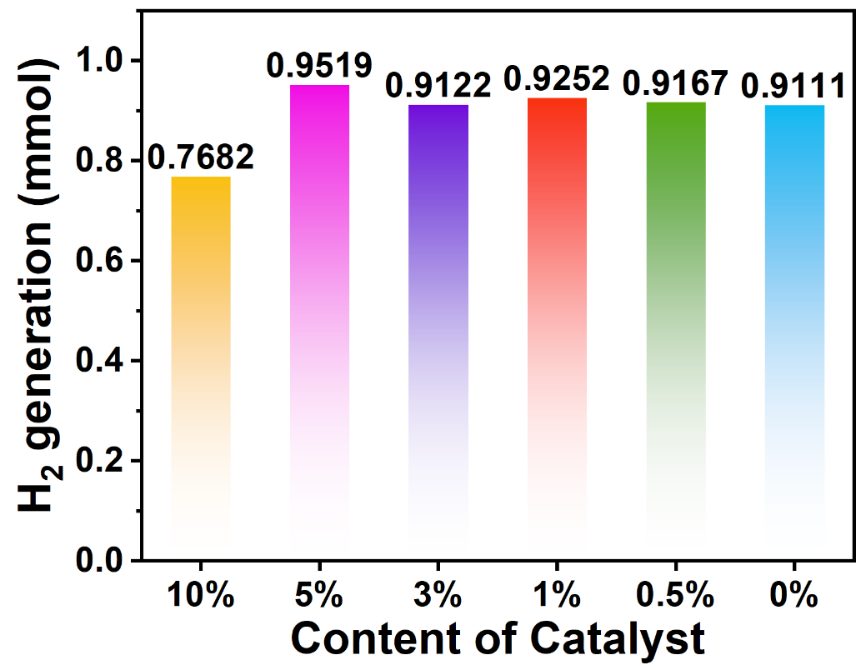


**Supplementary Figure 10.** Visible-light-driven hydrogen release capacities of AlH_3_-MOF and ball-milled AlH_3_ under visible light irradiation.


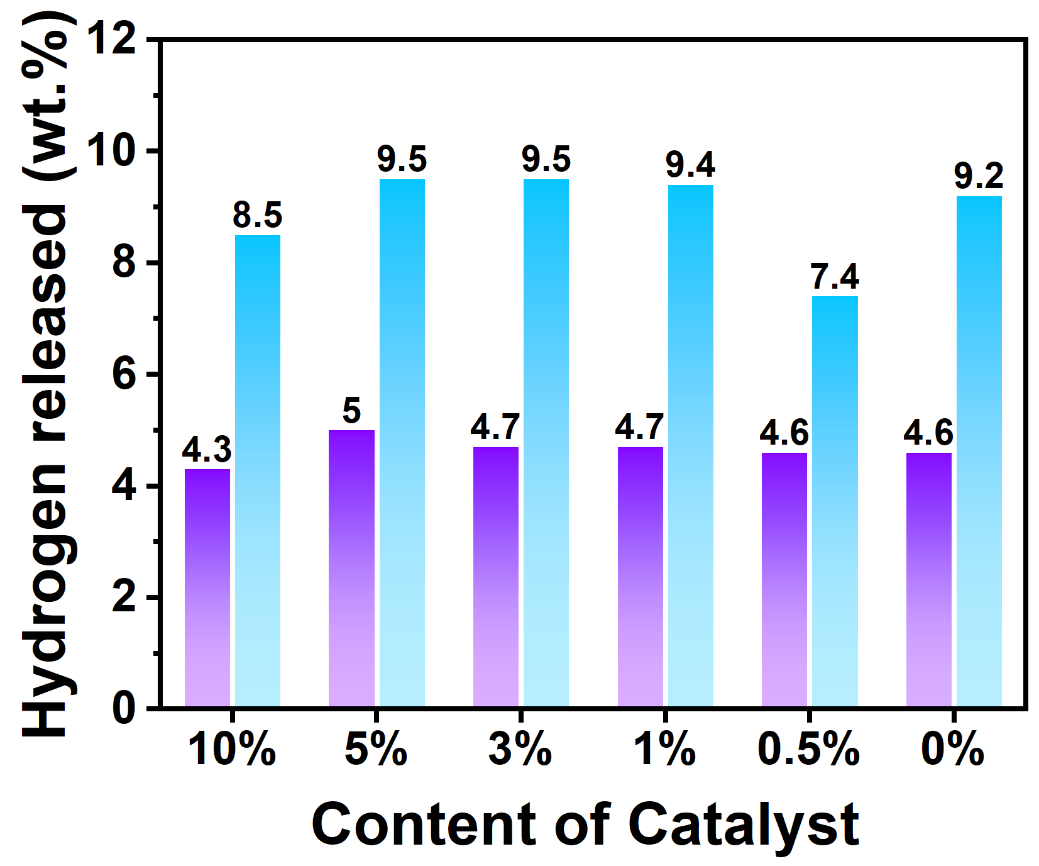


**Supplementary Figure 11.** Thermal-driven and visible-light-driven hydrogen release capacities of AlH_3_-MOF and ball-milled AlH_3_. Purple and blue are visible-light-driven hydrogen release capacity and thermal-driven hydrogen release capacity, respectively.


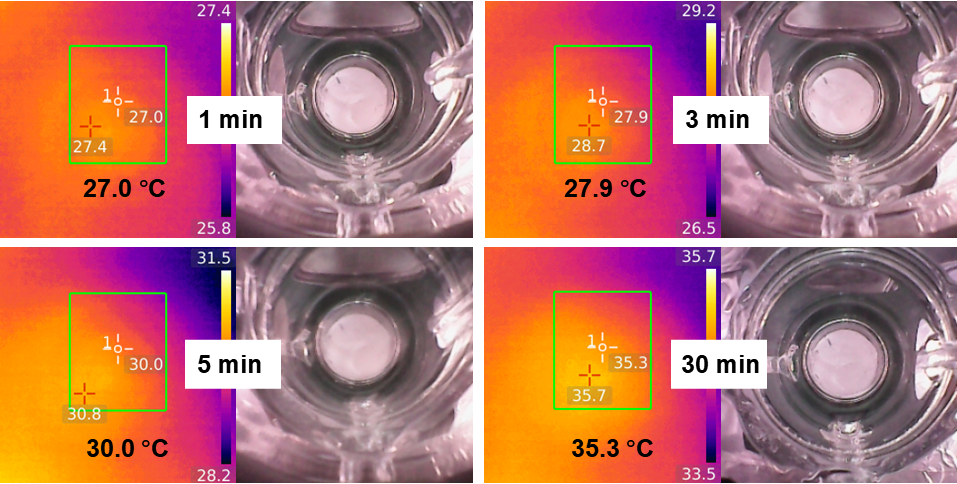


**Supplementary Figure 12.** *In-situ* detection of surface temperature of AlH_3_-MOF (1%) recorded by an infrared thermal imager under visible light irradiation (0.37 W/cm^2^) and with 25 °C circulating water-cooling system.

As shown in Supplementary Figure 12, the surface temperature of AlH_3_-MOF (1%) rises rapidly upon initial illumination, then exhibits attenuated heating due to active cooling from the circulating water system. After 30 minutes of visible light irradiation, the surface temperature of AlH_3_-MOF (1%) only reached 35.3 °C, which is far lower than the thermal-drive initial hydrogen release temperature of the material. Meanwhile, thermal homogeneity is maintained throughout the composite under visible light irradiation. This is sufficient to illustrate that photocatalysis still holds a dominant position during the dehydrogenation process.


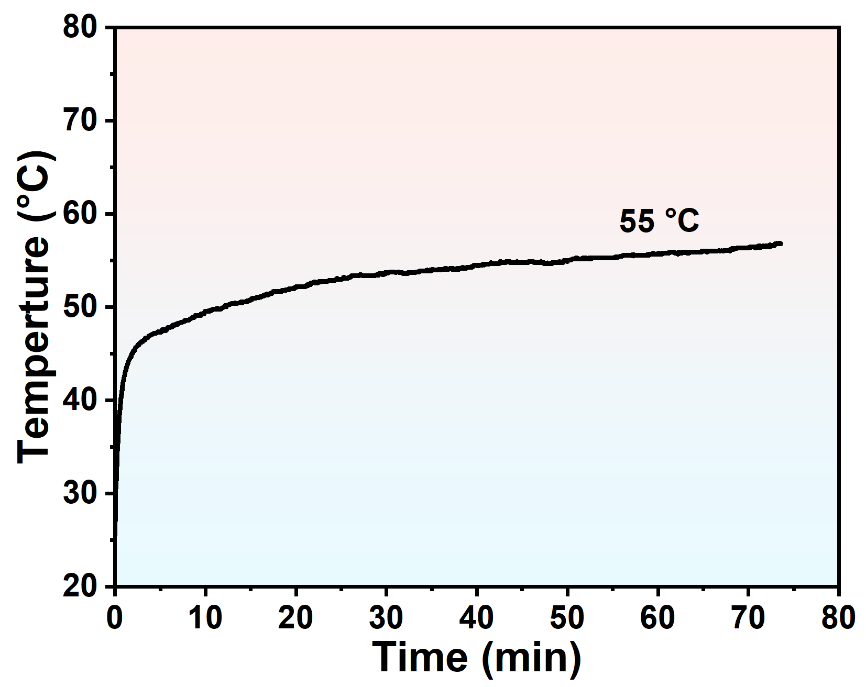


**Supplementary Figure 13.** AlH_3_-MOF (1%) surface temperature under visible light irradiation (0.37 W/cm^2^) and without active water-cooling.


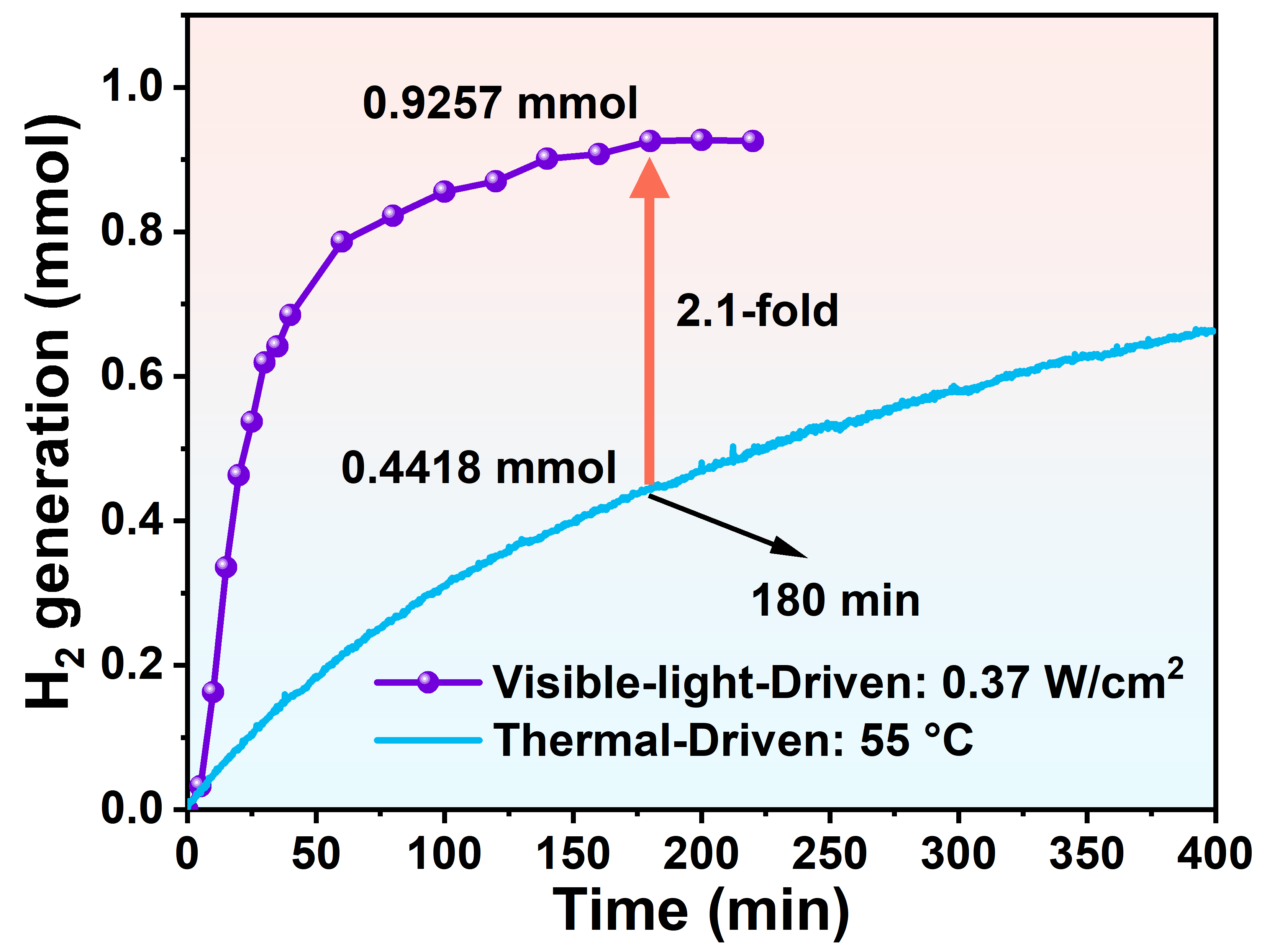


**Supplementary Figure 14.** Visible-light-driven (0.37 W/cm^2^) hydrogen release without active water-cooling and isothermal hydrogen release profile at 55 °C of AlH_3_-MOF (1%), respectively.


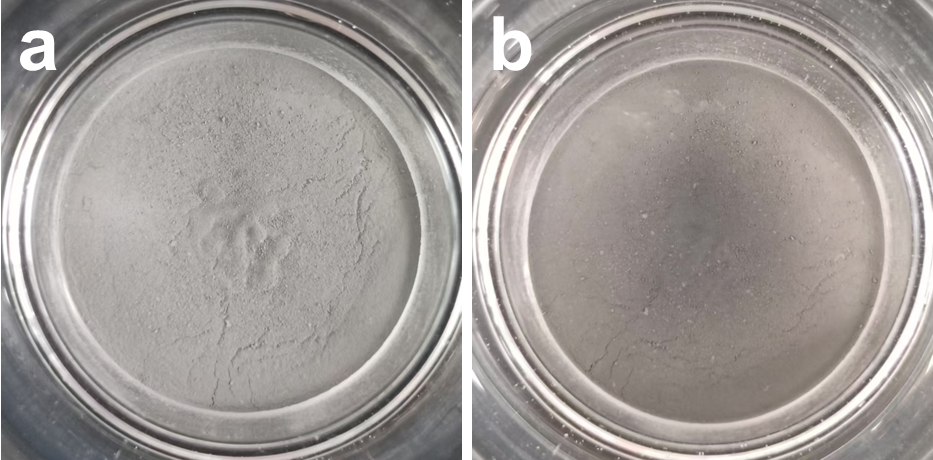


**Supplementary Figure 15.** Digital images of AlH_3_-MOF (1%) **a** before and **b** after visible-light-driven photocatalytic dehydrogenation (0.37 W/cm^2^) at room temperature.


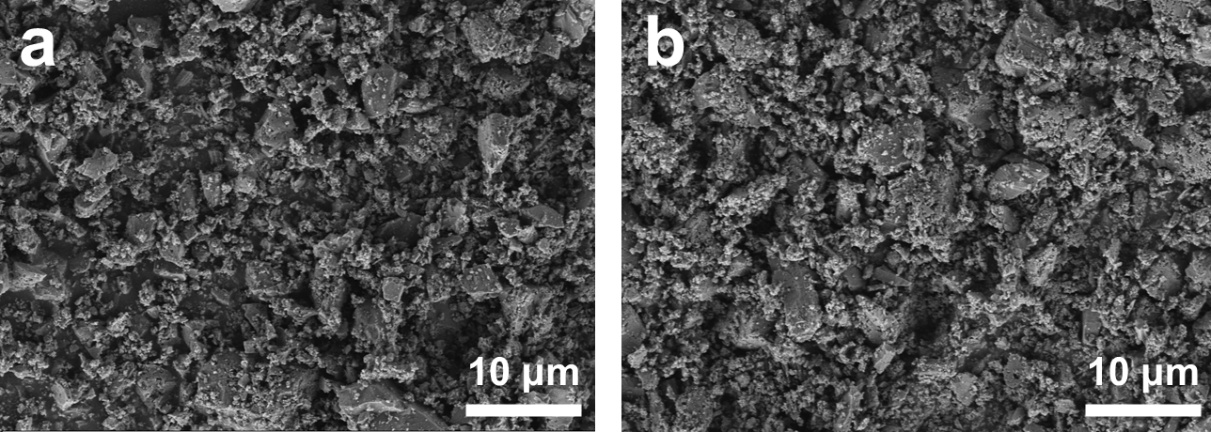


**Supplementary Figure 16.** SEM images of AlH_3_-MOF (1%) **a** before and **b** after visible-light-driven photocatalytic dehydrogenation (0.37 W/cm^2^) at room temperature.


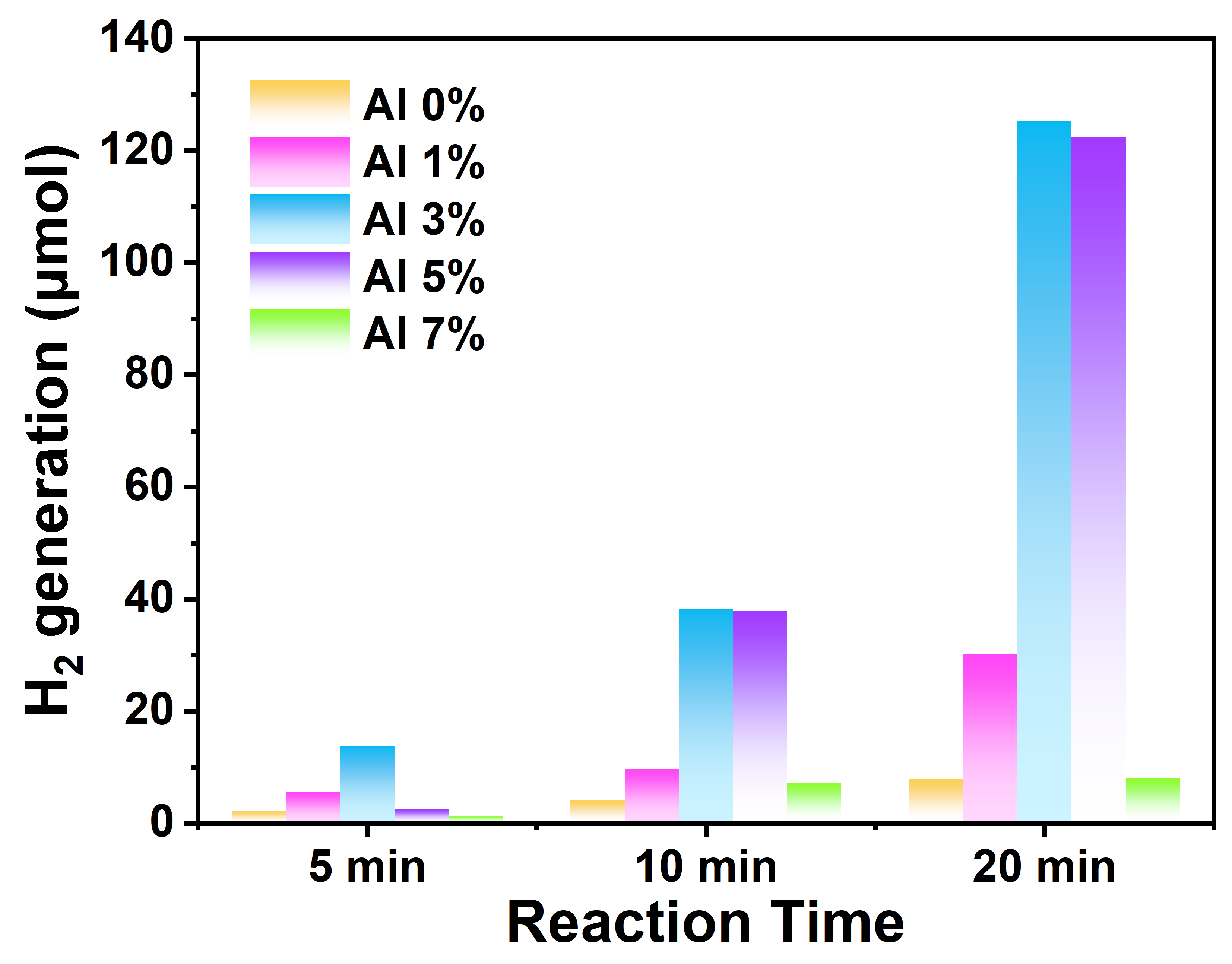


**Supplementary Figure 17.** Visible-light-driven hydrogen releases amounts of Al-AlH_3_-MOF at different reaction times under visible light irradiation (0.37 W/cm^2^) and at room temperature.


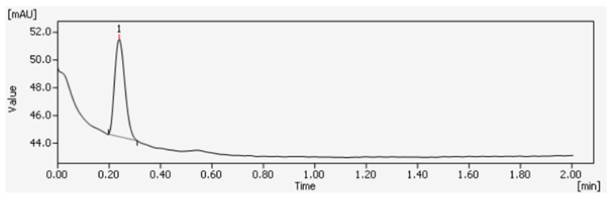


**Supplementary Figure 18.** Gas chromatographic analysis of photocatalytic dehydrogenation products from AlH_3_-MOF.

Gas chromatography (GC) conclusively verifies product purity, the chromatograms exclusively display H_2_ peaks under photocatalytic dehydrogenation process, with no detectable signals for O_2_, N_2_, or CO_2_ (Supplementary Figure 18). This confirms high-purity hydrogen evolution. Furthermore, all MOF substrates underwent rigorous gas-exchange pretreatment, involving 24-hour vacuum degassing at 60 °C followed by transfer to an argon glovebox, to ensure complete elimination of adsorbed gases prior to ball milling. These protocols ensure no exogenous gas contamination during reactions.


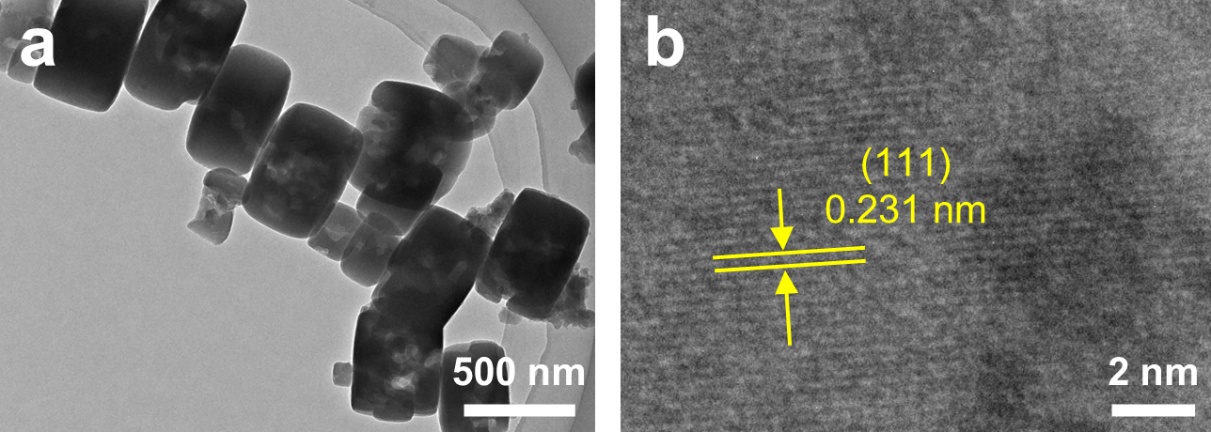


**Supplementary Figure 19.** **a** TEM and **b** HRTEM images with measured lattice spacing Al inset of Al/MOF.


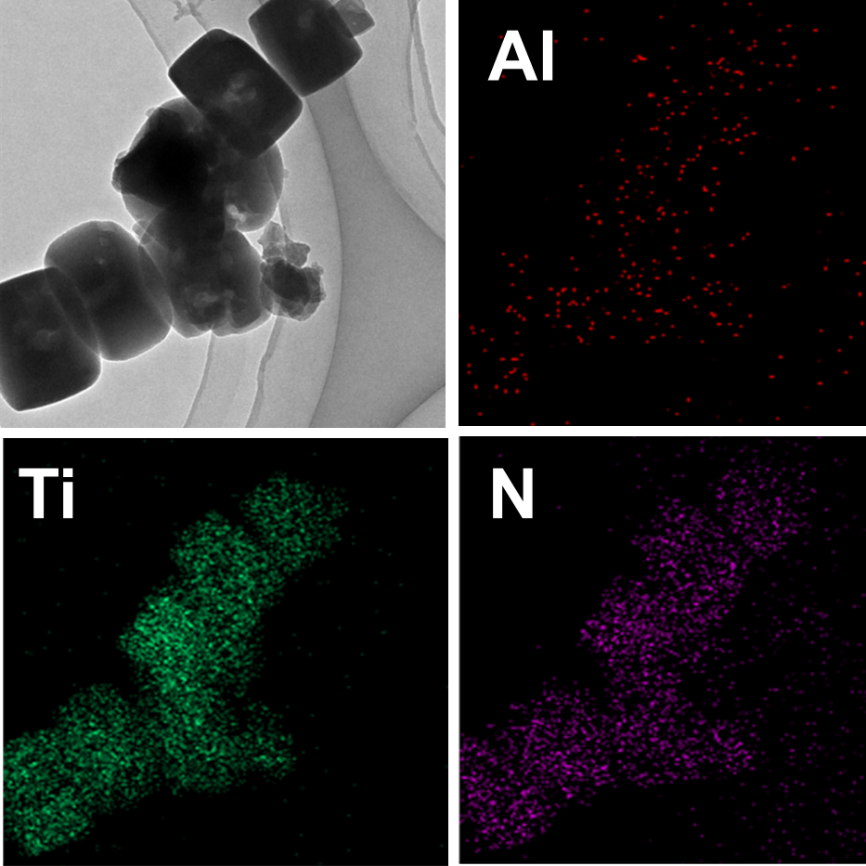


**Supplementary Figure 20.** TEM-EDS elemental mapping images of Al/MOF.


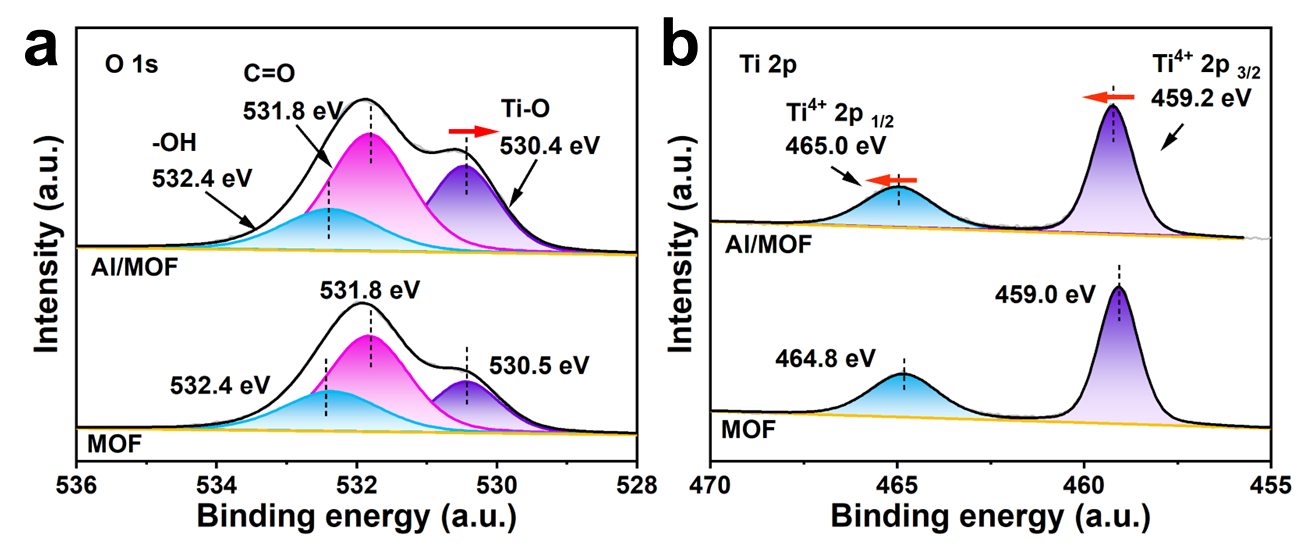


**Supplementary Figure 21.** High-resolution XPS spectra for **a** Ti 2*p* and **b** O 1*s* of Al/MOF, with MOF for comparison.


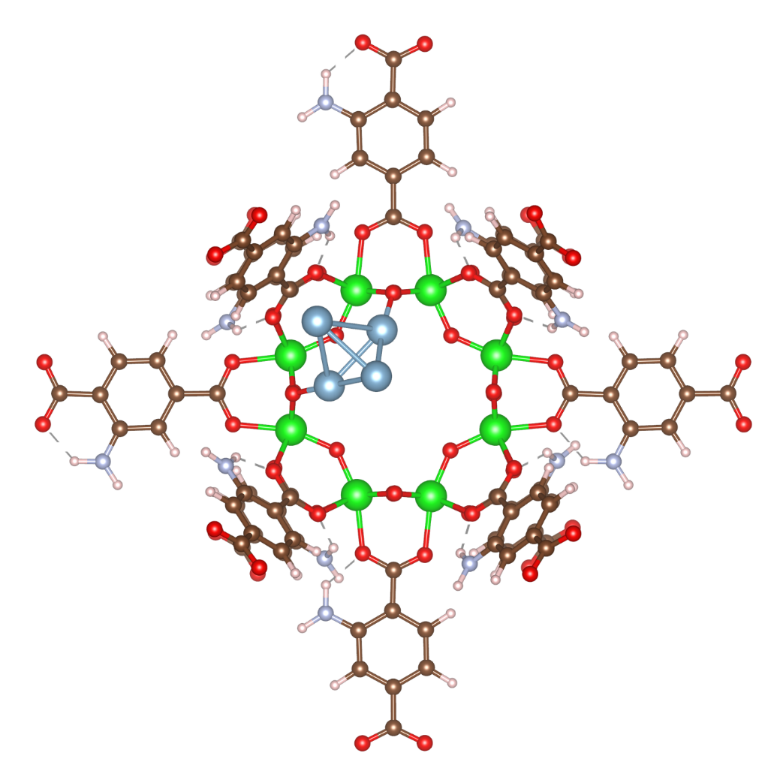


**Supplementary Figure 22.** Top view of optimized crystal model of Al/MOF.


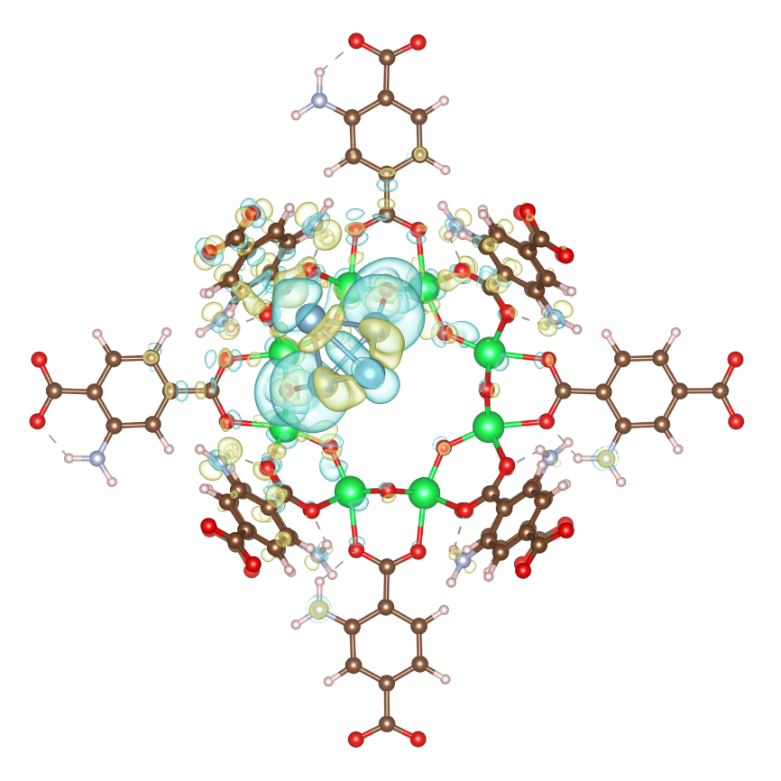


**Supplementary Figure 23.** Top view of the electron density difference maps of Al/MOF.


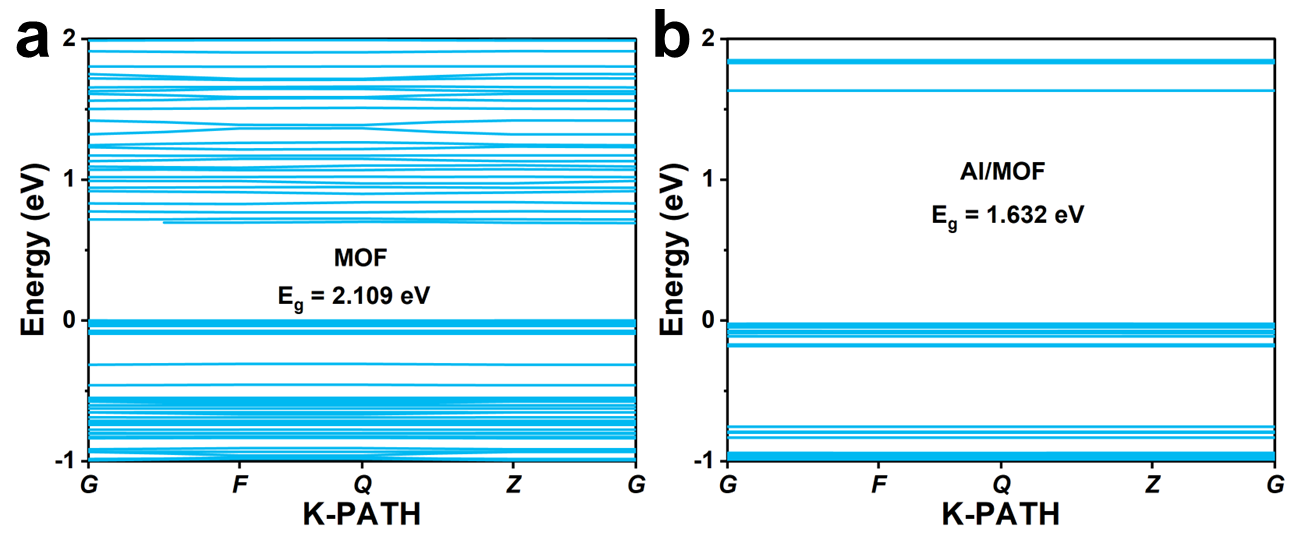


**Supplementary Figure 24.** Band structures of a MOF and b Al/MOF.


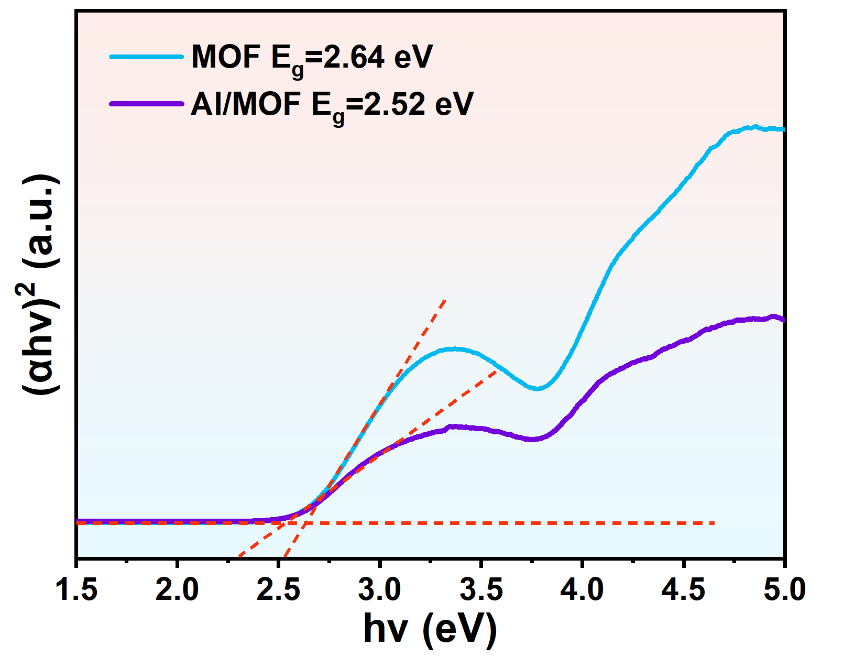


**Supplementary Figure 25.** Tauc plots for energy gap of Al/MOF and MOF.


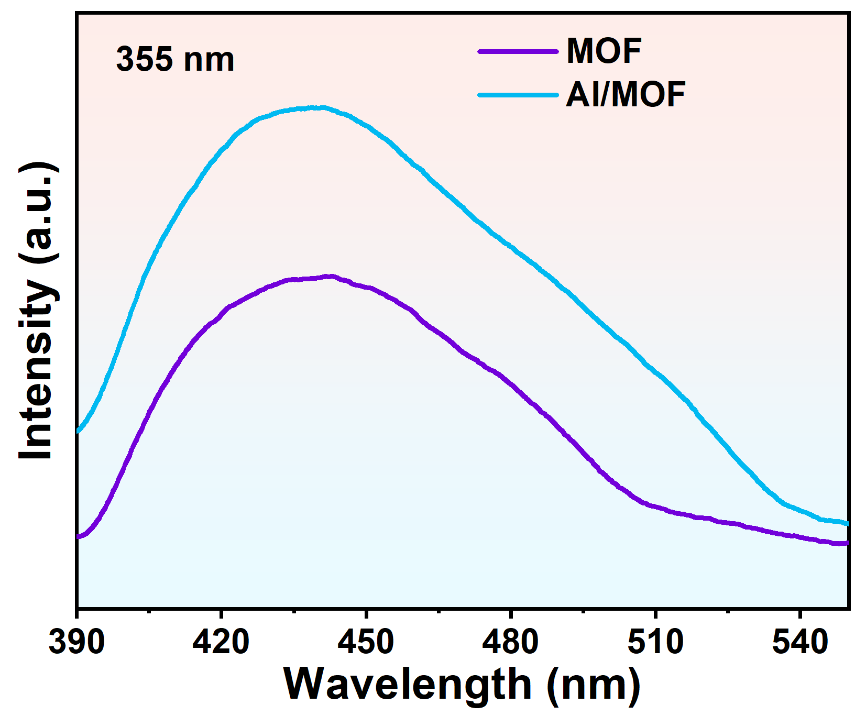


**Supplementary Figure 26.** Steady-state PL spectra of MOF and Al/MOF excited by 355 nm.


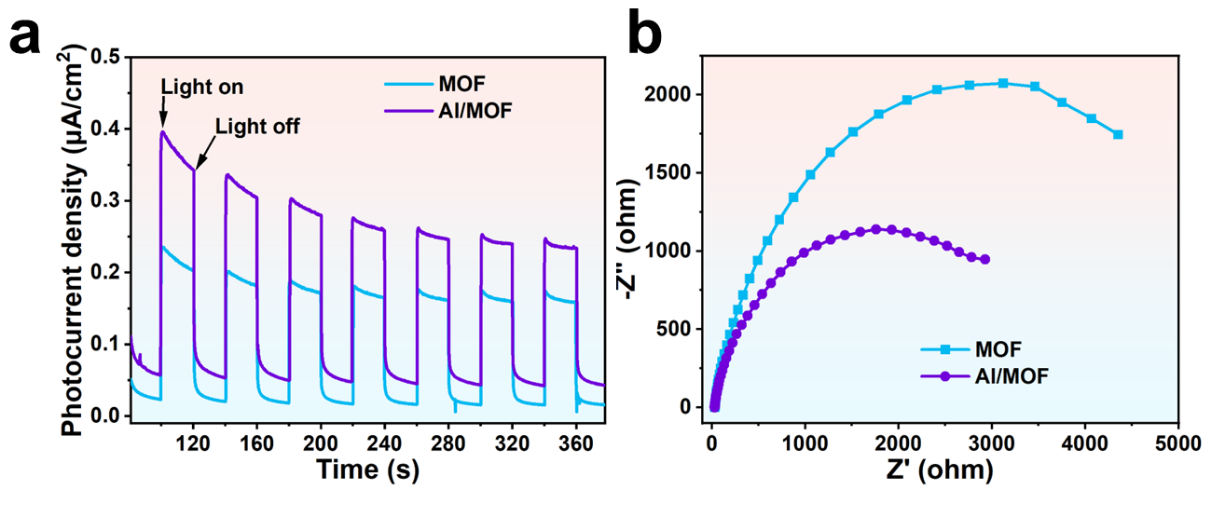


**Supplementary Figure 27. a** Transient photocurrent responses and **b** EIS Nyquist plots of MOF and Al/MOF.


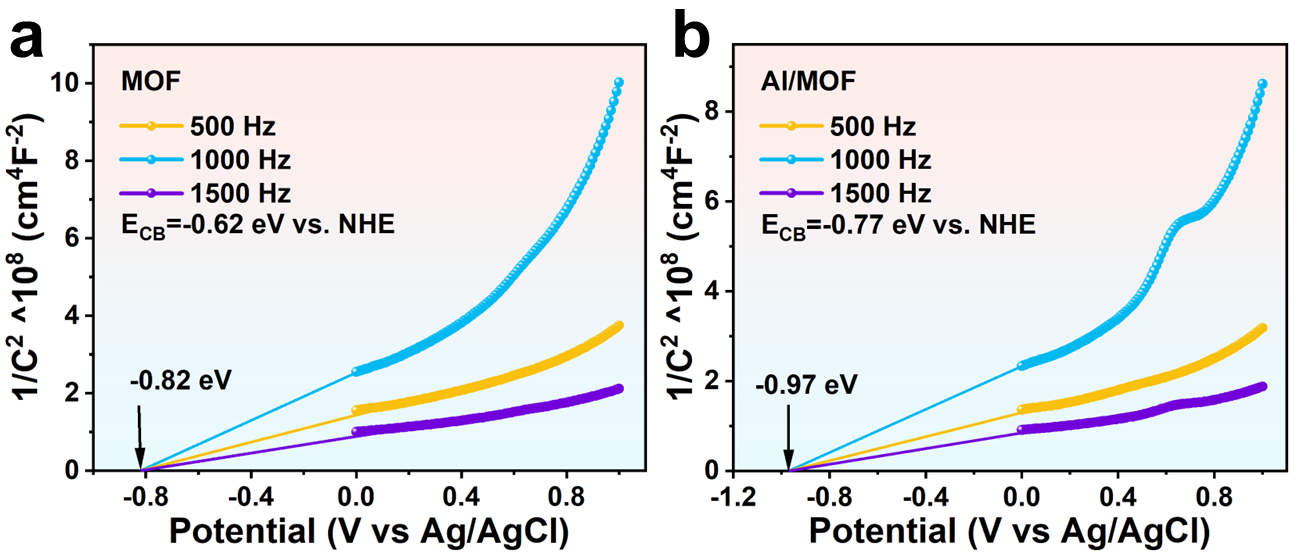


**Supplementary Figure 28.** Mott-Schottky plots with different frequencies of **a** MOF and **b** Al/MOF.


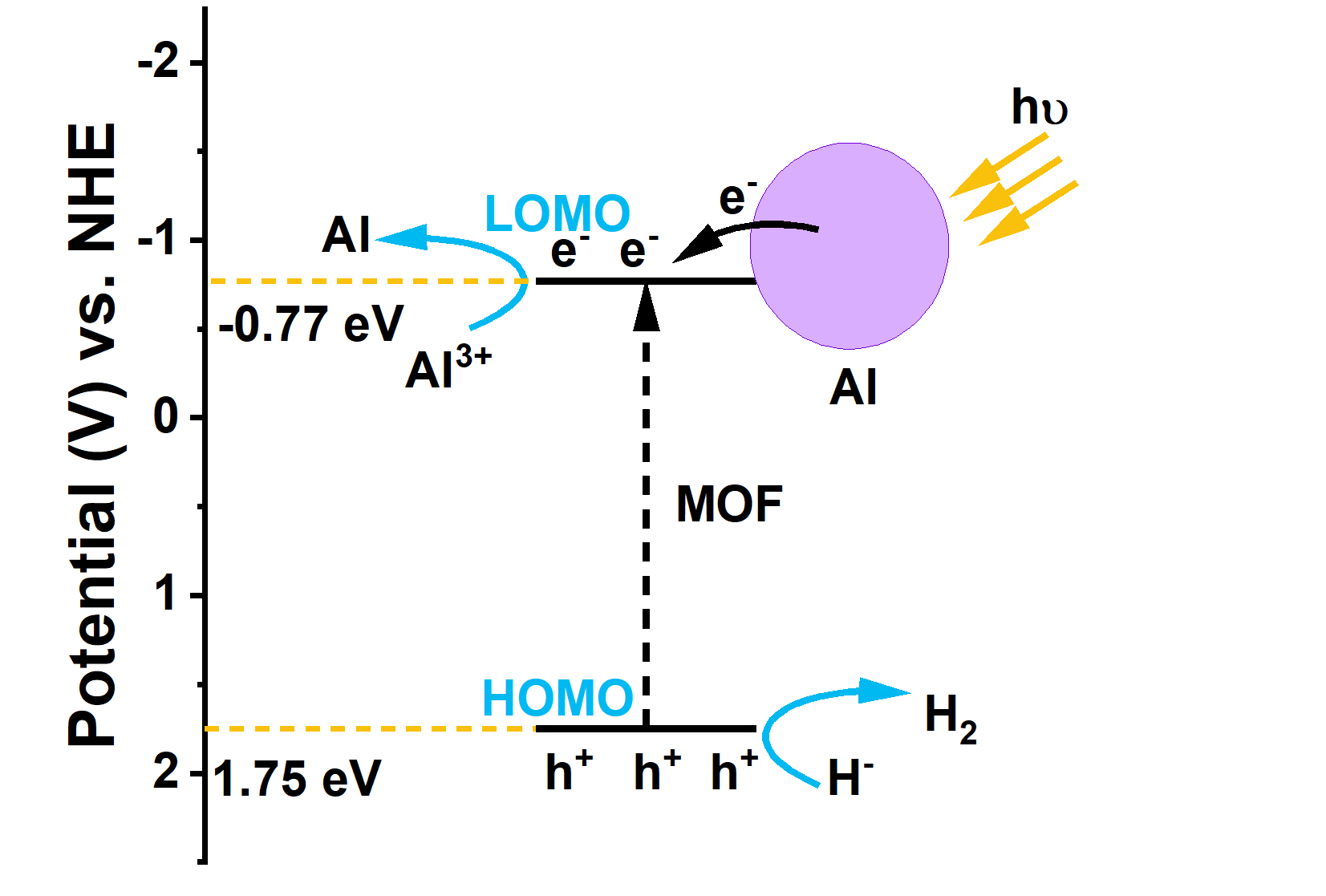


**Supplementary Figure 29.** Schematic illustration of charge transfer and photocatalytic H_2_ evolution path of AlH_3_- Al/MOF.


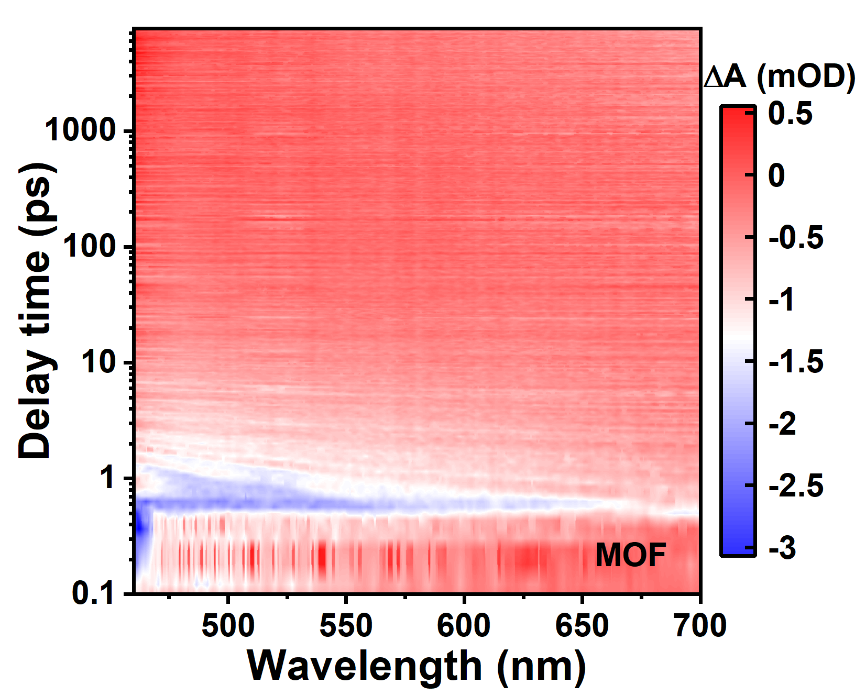


**Supplementary Figure 30.** Fs-TA in contour maps of MOF.


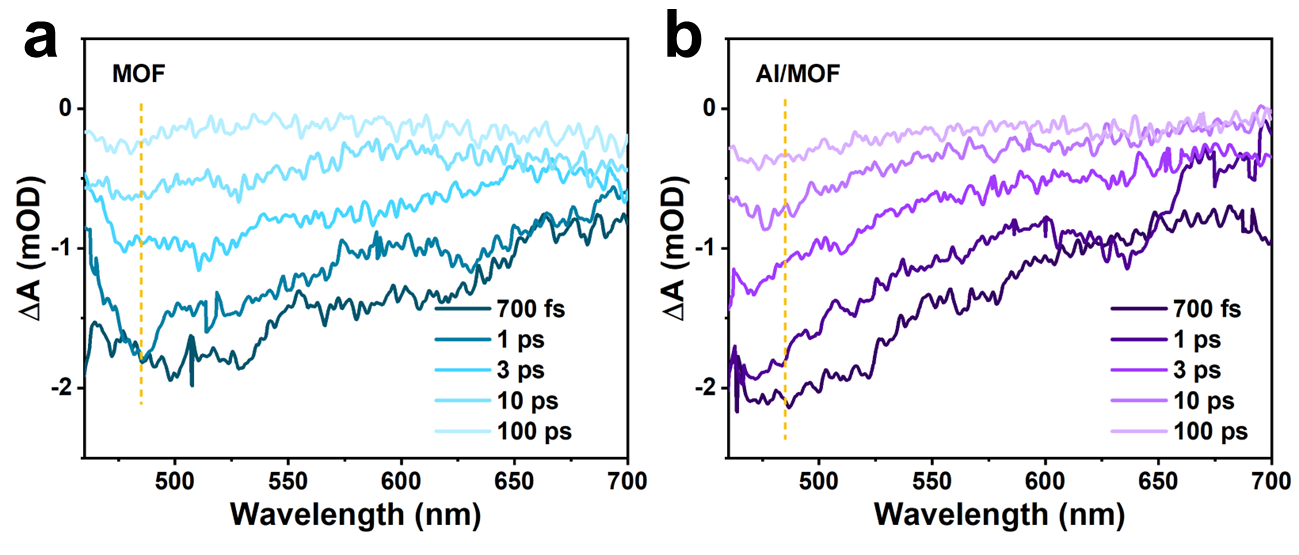


**Supplementary Figure 31.** Fs-TA spectra at selected pump-probe delays of **a** MOF and **b** Al/MOF.


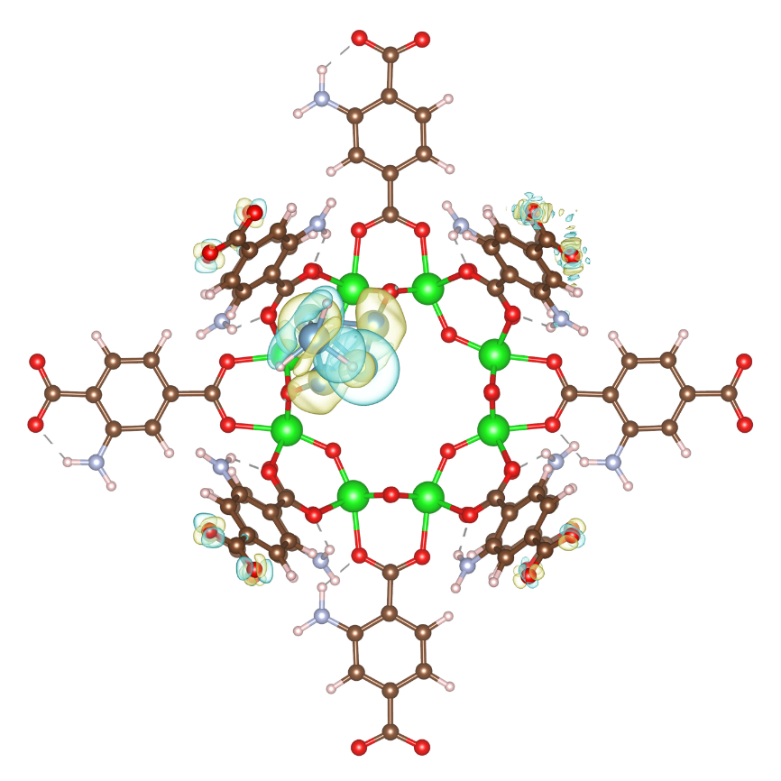


**Supplementary Figure 32.** Top view of the electron density difference maps of AlH_3_-Al/MOF.


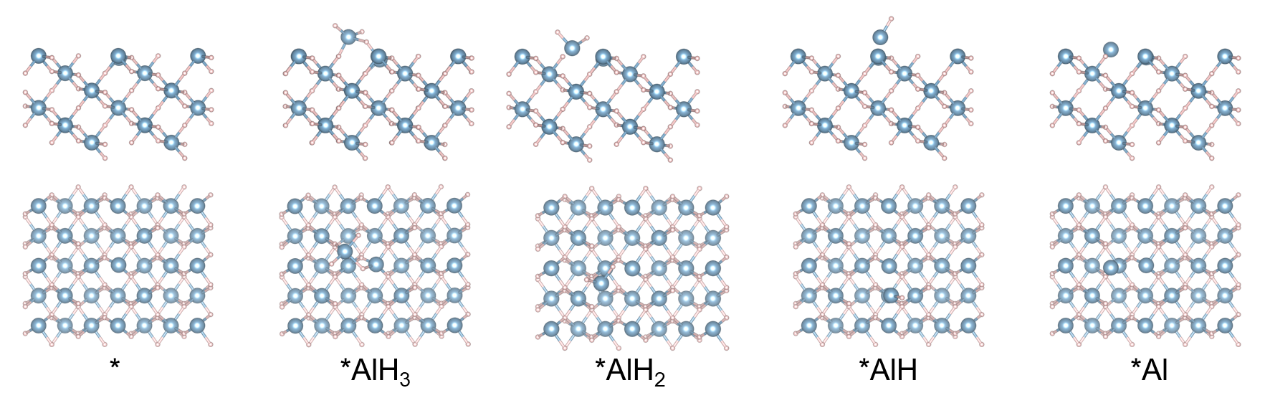


**Supplementary Figure 33.** Intermediates and surface configurations during AlH_3_ dehydrogenation process.


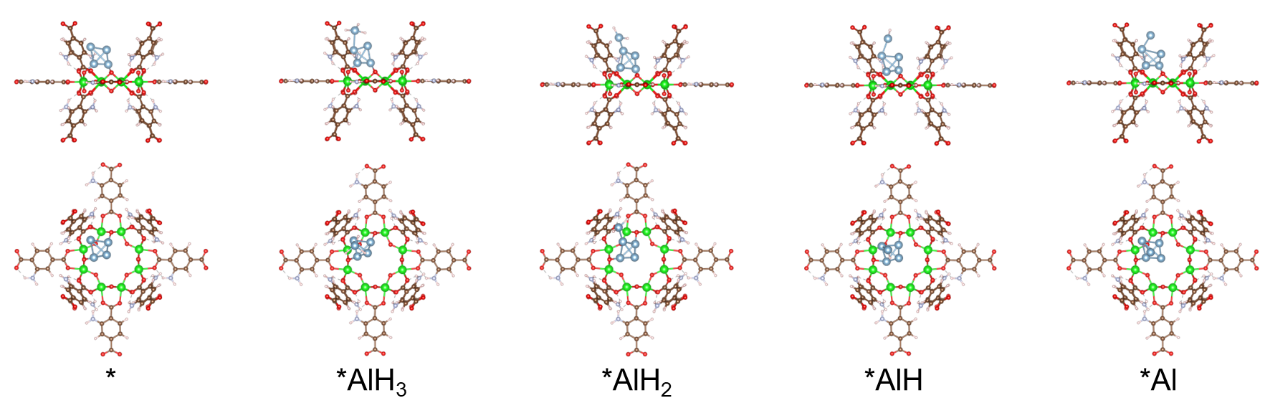


**Supplementary Figure 34.** Intermediates and surface configurations during AlH_3_-Al/MOF dehydrogenation process.

## Supplementary Tables

**Supplementary Table 1.** XPS results for Al 2*p* (100%)

| Atomic orbital | Ball-milled AlH_3_ | AlH_3_-MOF (1%) | DH-AlH_3_-MOF (1%) |
| --- | --- | --- | --- |
| Al^3+^ | 100% | 95.54% | 76.26% |
| Al^0^ |  | 4.46% | 23.74% |

**Supplementary Table 2.** XPS results for Ti 2*p* (100%)

| Atomic orbital | MOF | AlH_3_-MOF (1%) | DH-AlH_3_-MOF (1%) |
| --- | --- | --- | --- |
| Ti^4+^ 2*p*_1/2_ | 34.95% | 37.3% | 20.83% |
| Ti^3+^ 2*p*_1/2_ |  | 3.79% | 12.5% |
| Ti^4+^ 2*p*_3/2_ | 65.05% | 51.27% | 41.65% |
| Ti^3+^ 2*p*_3/2_ |  | 7.64% | 25.02% |

**Supplementary Table 3.** Thermal-driven and visible-light-driven hydrogen release capacities for AlH_3_-MOF and ball-milled AlH_3_.

| AlH_3_-MOF (x%) | 10 | 5 | 3 | 1 | 0.5 | Ball-milled AlH_3_ |
| --- | --- | --- | --- | --- | --- | --- |
| Thermal-driven hydrogen release capacity (wt.%) | 8.5 | 9.5 | 9.5 | 9.4 | 7.4 | 9.2 |
| Visible-light-driven hydrogen release capacity (wt.%) | 4.3 | 5.0 | 4.7 | 4.7 | 4.6 | 4.6 |
